# Reconstructing the Evolution of *Brachypodium* Genomes Using Comparative Chromosome Painting

**DOI:** 10.1371/journal.pone.0115108

**Published:** 2014-12-10

**Authors:** Alexander Betekhtin, Glyn Jenkins, Robert Hasterok

**Affiliations:** 1 Department of Plant Anatomy and Cytology, Faculty of Biology and Environmental Protection, University of Silesia in Katowice, Katowice, Poland; 2 Institute of Biological, Environmental and Rural Sciences, Edward Llwyd Building, Aberystwyth University, Penglais, Aberystwyth, Ceredigion, Wales, United Kingdom; University of Massachusetts Amherst, United States of America

## Abstract

*Brachypodium distachyon* is a model for the temperate cereals and grasses and has a biology, genomics infrastructure and cytogenetic platform fit for purpose. It is a member of a genus with fewer than 20 species, which have different genome sizes, basic chromosome numbers and ploidy levels. The phylogeny and interspecific relationships of this group have not to date been resolved by sequence comparisons and karyotypical studies. The aims of this study are not only to reconstruct the evolution of *Brachypodium* karyotypes to resolve the phylogeny, but also to highlight the mechanisms that shape the evolution of grass genomes. This was achieved through the use of comparative chromosome painting (CCP) which hybridises fluorescent, chromosome-specific probes derived from *B. distachyon* to homoeologous meiotic chromosomes of its close relatives. The study included five diploids (*B. distachyon* 2n = 10, *B. sylvaticum* 2n = 18, *B. pinnatum* 2n = 16; 2n = 18, *B. arbuscula* 2n = 18 and *B. stacei* 2n = 20) three allotetraploids (*B. pinnatum* 2n = 28, *B. phoenicoides* 2n = 28 and *B. hybridum* 2n = 30), and two species of unknown ploidy (*B. retusum* 2n = 38 and *B. mexicanum* 2n = 40). On the basis of the patterns of hybridisation and incorporating published data, we propose two alternative, but similar, models of karyotype evolution in the genus *Brachypodium*. According to the first model, the extant genome of *B. distachyon* derives from *B. mexicanum* or *B. stacei* by several rounds of descending dysploidy, and the other diploids evolve from *B. distachyon* via ascending dysploidy. The allotetraploids arise by interspecific hybridisation and chromosome doubling between *B. distachyon* and other diploids. The second model differs from the first insofar as it incorporates an intermediate 2n = 18 species between the *B. mexicanum* or *B. stacei* progenitors and the dysploidic *B. distachyon*.

## Introduction

The Poaceae is one of the largest families of flowering plants, with over 10,000 species spread widely throughout the earth in different climatic zones, and is an important component of most land ecosystems. The extraordinary diversity of morphological and physiological characteristics and efficient propagation mechanisms of grasses have ensured their evolutionary success in virtually every habitat. Some important members of this group contribute to more than 60% of global food production [1]. The ecological and economic significance of the grasses has resulted in their frequent scientific scrutiny, for example through the application of cytogenetics and more recently comparative genetics and genomics.

In comparison with other groups of organisms, plant nuclear genomes show exceptional plasticity in terms of DNA contents, and number, size and shape of chromosomes. These features are subject to evolutionary changes, and may differ greatly even amongst closely related species [2]. There are many mechanisms that shape to different extents the structure of karyotypes in plants. One of the most spectacular involves changes in chromosome number, which may affect both entire chromosome sets (polyploidy) and individual chromosomes (aneuploidy and dysploidy). Inter- and intrachromosomal rearrangements, such as translocations, fusions and fissions can also contribute to changes in chromosome number, whilst insertions, duplications, inversions and in some cases deletions usually act as minor agents of genome shuffling (for recent review, see [3]). To date, most of the information about the evolutionary forces which have shaped the structure of extant grass genomes comes from *in silico* archeogenomic studies of DNA sequences [4]. Recent technological advances, such as high-throughput DNA sequencing, have enabled high-resolution comparative genomics and bioinformatic analyses. More than 15 plant genomes have currently been sequenced which offers the opportunity not only to compare the organisation of modern genomes but also to infer their evolutionary history from *in silico* ‘reconstruction’ of their putative ancestors [4], [5].

There are several models of grass genome evolution which are linked with karyotypes. One of the first and best known is the ‘crop circle’ of Moore and co-workers which shows that the structure of the genomes of several major grass species can be described in terms of the rearrangement of relatively few conserved chromosomal blocks of rice, together with various polyploidisation and diploidisation events [6], [7]. Complex studies of paleoduplications of thousands of orthologues and paralogues forms the basis of a widely accepted model that explains the evolution of grass genomes at the level of the chromosome from a common ancestor with a genome of at least 33 Mb in size, comprising most likely either five [8]–[10] or seven [4] protochromosomes. According to this model, this protoancestor underwent paleotetraploidisation followed by reciprocal translocations that led to a 12-chromosome intermediate. Interestingly, because of the availability of genomic data it has been shown that the general ‘landscape’ of karyotype structure and evolution of angiosperms is very similar for most angiosperms. All monocot and eudicot genomes analysed so far can be reconstructed from putative intermediate ancestral genomes containing 12 (monocots) and 21 (eudicots) protochromosomes, implying that all angiosperms are in fact ancient polyploids. The corollary is that the large differences in chromosome numbers of modern species have resulted from various and more recent family- and lineage-specific reorganisation and polyploidisation events [4].

Some recent studies of eudicots have effectively combined resources resulting from whole genome sequencing (WGS) projects with advanced cytomolecular mapping. A good example is the use of BAC (Bacterial Artificial Chromosome) vectors containing large genomic DNA inserts as informative, chromosome- and region-specific probes to physically map pachytene or somatic chromosomes using cross-species fluorescence *in situ* hybridisation (FISH). This approach has the advantage of enabling direct visualisation and observation of chromosomal rearrangements involved in karyotype differentiation in related genomes. It has been used effectively to study fine-scale chromosome rearrangements and karyotype evolution in *Solanum* [11] and some other genera of Solanaceae [12], and has potential utility as a diagnostic tool in introgression breeding [13], [14]. Similar studies have been reported in a few other plant genera such as *Phaseolus* [15], [16] and *Brachypodium* [17]–[20], underpinning syntenic and molecular phylogenetic analyses and facilitating the integration of physical, genetic and cytogenetic maps.

Chromosome painting (CP) or chromosome *in situ* suppression is one of the most effective and informative tools of modern molecular cytogenetics, enabling selective visualisation of entire chromosomes or their segments through the use of FISH with chromosome-specific DNA probes [21]. Until recently, this approach was used only to study animal chromosomes in the contexts of the molecular cytotaxonomy of primates [22], the structural and functional compartmentalisation of the nucleus [23] and clinical diagnostics of chromosomal aberrations linked with various human diseases [24], [25]. Though technically more demanding in plants, CP was first used successfully in *Arabidopsis thaliana* [26] following the publication of its genomic sequence [27]. Later studies of comparative chromosome painting (CCP) in close crucifer relatives gave unprecedented insight into the evolution of their genomes at the chromosomal level, enabling a description of mechanisms of descending dysploidy in this group of species [28]–[32]. Recently, a technically novel, single-copy, gene-based comparative CP (CCP) approach has enabled effective analysis of chromosome rearrangements in several species of *Cucumis* [33].

Although rice is a model monocot with well-established genomic infrastructure and long-published genomic sequence [34], no CP has been undertaken in this species. By contrast, the temperate grass model, *Brachypodium distachyon,* is the first monocot painted [35] as a result of its advanced genomic infrastructure, such as BAC DNA libraries and bioinformatic data generated by its WGS project [18], [19], combined with its well-developed cytogenetic platform [36]–[38]. The genus *Brachypodium* comprises 14–19 species of different genome sizes and complexities and includes diploids with chromosome base numbers of 5, 8, 9 and 10 [39] as well as allopolyploids with 2n = 28, 38 [40], [41]. Although some studies have addressed the phylogenetic relations within this genus [17], [42], [43], there are still many uncertainties about both the taxonomic identity of some allopolyploids and the karyological evolution of the different *Brachypodium* species. More importantly, the genus *Brachypodium* has not been exploited yet to the full as an excellent model system for studying karyotype evolution and divergence in grasses. The CCP approach, based on ordered BAC pools derived from the *B. distachyon* genome, has so far been limited to only three species, i.e. *B. distachyon* (2n = 10), *B. stacei* (2n = 20) and *B. hybridum* (2n = 30) [35]. In the present study, the karyotype organisation of nine *Brachypodium* species is compared and the potential models of intrageneric genome evolution are discussed.

## Results

Pools of 142, 55, 96, 59 and 23 low-repeat BAC clones derived from *B. distachyon* chromosomes (Bd1–Bd5) were used for CCP of homoeologous chromosomes of both diploid and allotetraploid *Brachypodium* species, as well as *B. mexicanum* and *B. retusum* whose ploidy status is unclear. As the pattern of CCP within the diploids 2n = 16; 18 (with exception of *B. stacei*) and allopolyploids 2n = 28 (except *B. hybridum*) showed only minor differences, the photomicrographs show results only for one representative species within a group. In all experiments, the probes derived from the *B. distachyon* short arm (designated Bd1-5S) and long arm (designated Bd1-5L) were visualised by green fluorescence and red fluorescence, respectively. The experiments were performed with one chromosome-specific BAC pool per slide, as the simultaneous use of probes from more than one chromosome caused massive cross-hybridisation. Reprobing the same slides with different paints was not feasible due to the destructive action of pepsin, which was used to remove cytoplasm from the preparations.

### Bd1-derived pools of clones

**Figure 1:**
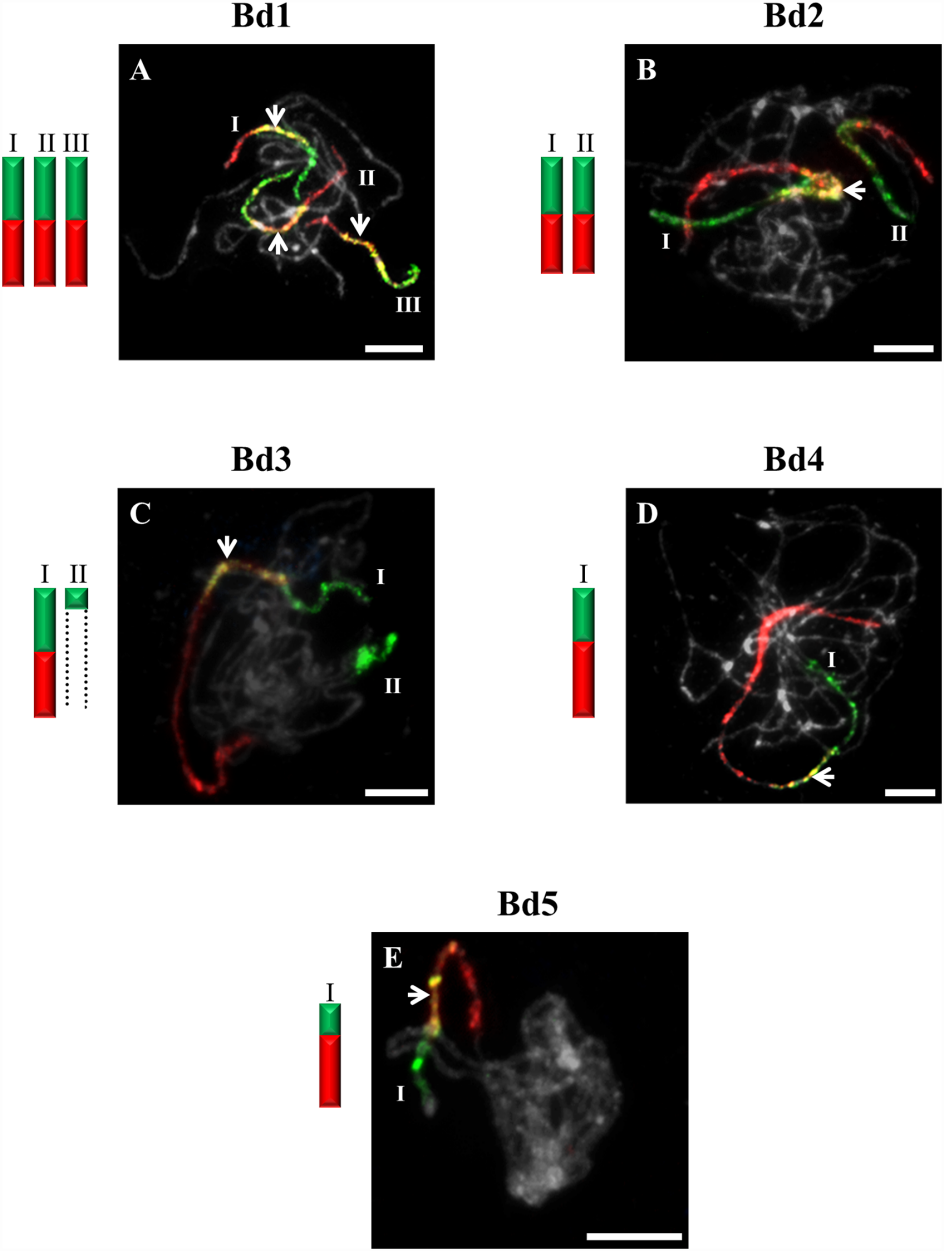
CCP in pachytene bivalents of *B. sylvaticum* (2n = 18) using BAC pools spanning the short (green fluorescence) and long (red fluorescence) arms of all five (Bd1-Bd5) *B. distachyon* chromosomes. Bd1- (**A**), Bd2- (**B**), Bd3- (**C**), Bd4- (**D**), Bd5-specific probes (**E**). Painted bivalents or their fragments in the photomicrographs are numbered arbitrarily using Roman numerals, which correspond to those on the idiograms. Bivalents that cannot be traced end- to-end are marked by dotted open-ended lines. White arrows indicate yellow fluorescence caused by the hybridisation of non-specific repeats common to the two chromosome arms. Chromosomes in Figures 1–7 are counterstained with DAPI. Relative lengths of chromosomes in the idiograms are only approximate. Bar: 10 µm.

**Figure 2:**
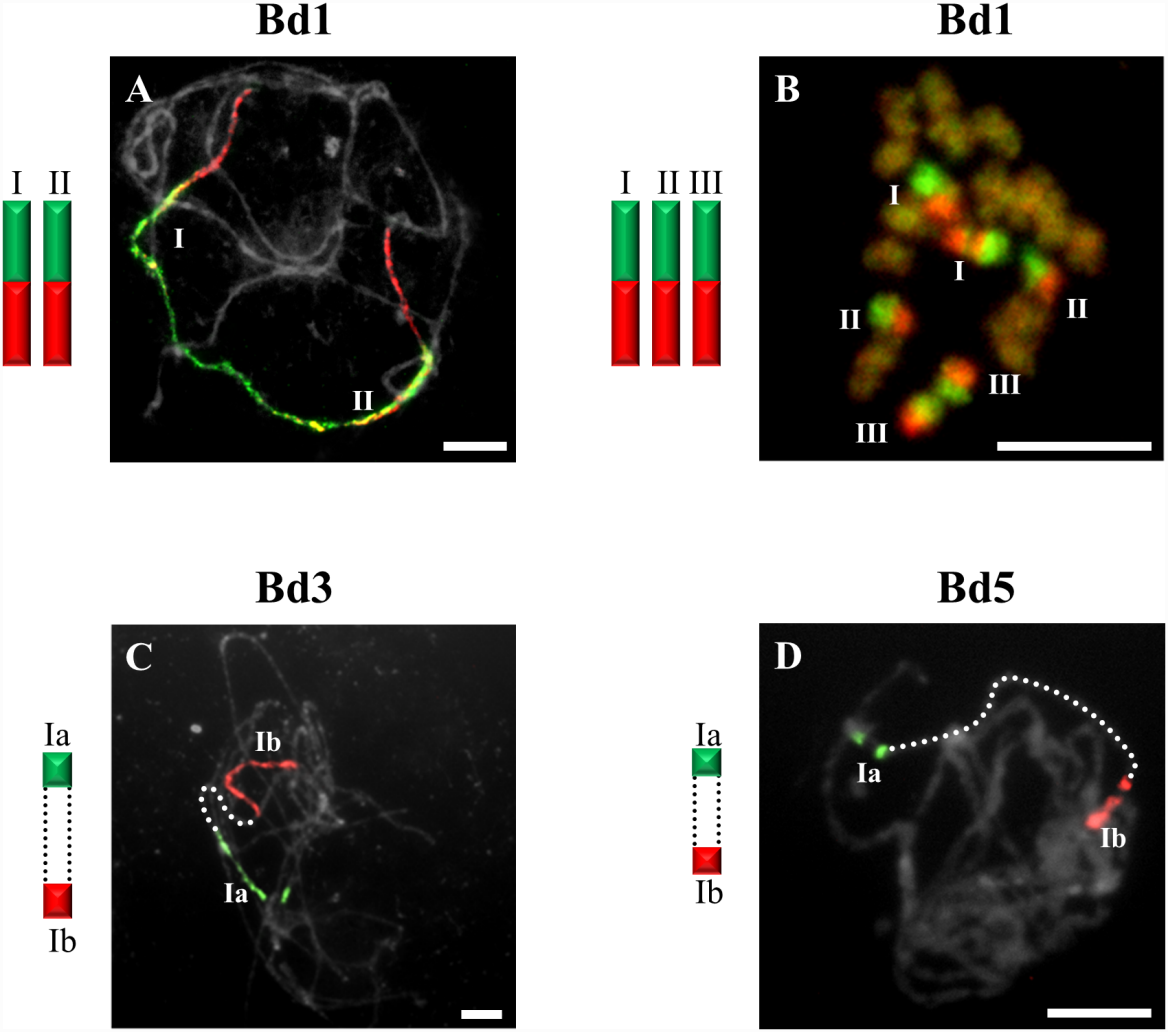
CCP of pachytene and somatic chromosomes showing key differences in the organisation of karyotypes of selected diploids with 2n = 16 and 2n = 18 using BAC pools spanning the short (green fluorescence) and long (red fluorescence) arms of *B. distachyon* chromosomes. Bd1 to *B. pinnatum* 2n = 16 (**A**), Bd1 to *B. pinnatum* 2n = 18 (**B**), Bd3 to *B. arbuscula* 2n = 18 (**C**) and Bd5 to *B. arbuscula* 2n = 18 (**D**). Bar: 10 µm

**Figure 3:**
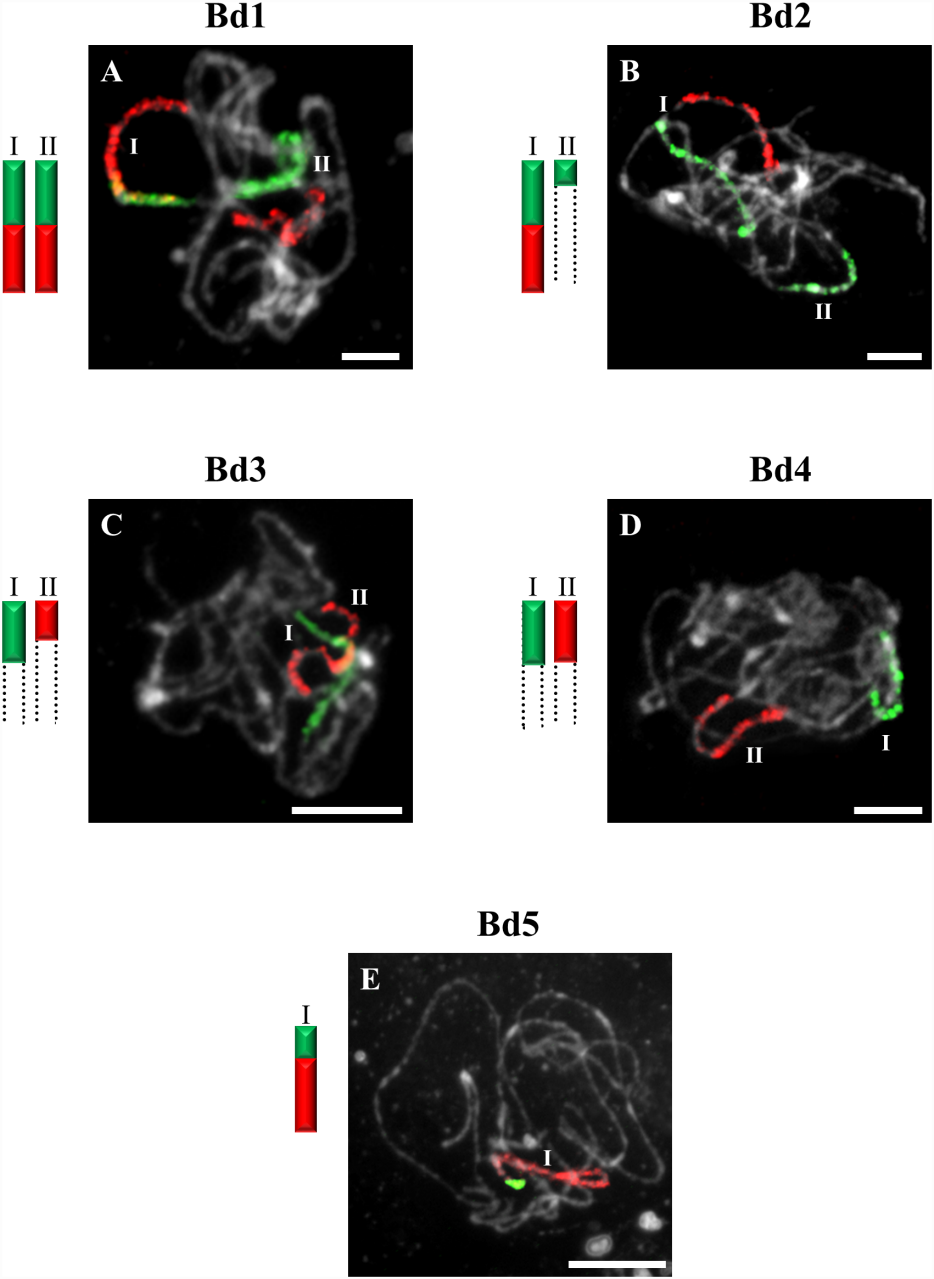
CCP in pachytene bivalents of *B. stacei* (2n = 20) using BAC pools spanning the short (green fluorescence) and long (red fluorescence) arms of all five (Bd1–Bd5) *B. distachyon* chromosomes. Bd1- (**A**), Bd2- (**B**), Bd3- (**C**), Bd4- (**D**), Bd5-specific probes (**E**). All other information as in Figure 1. Bar: 10 µm.

**Figure 4:**
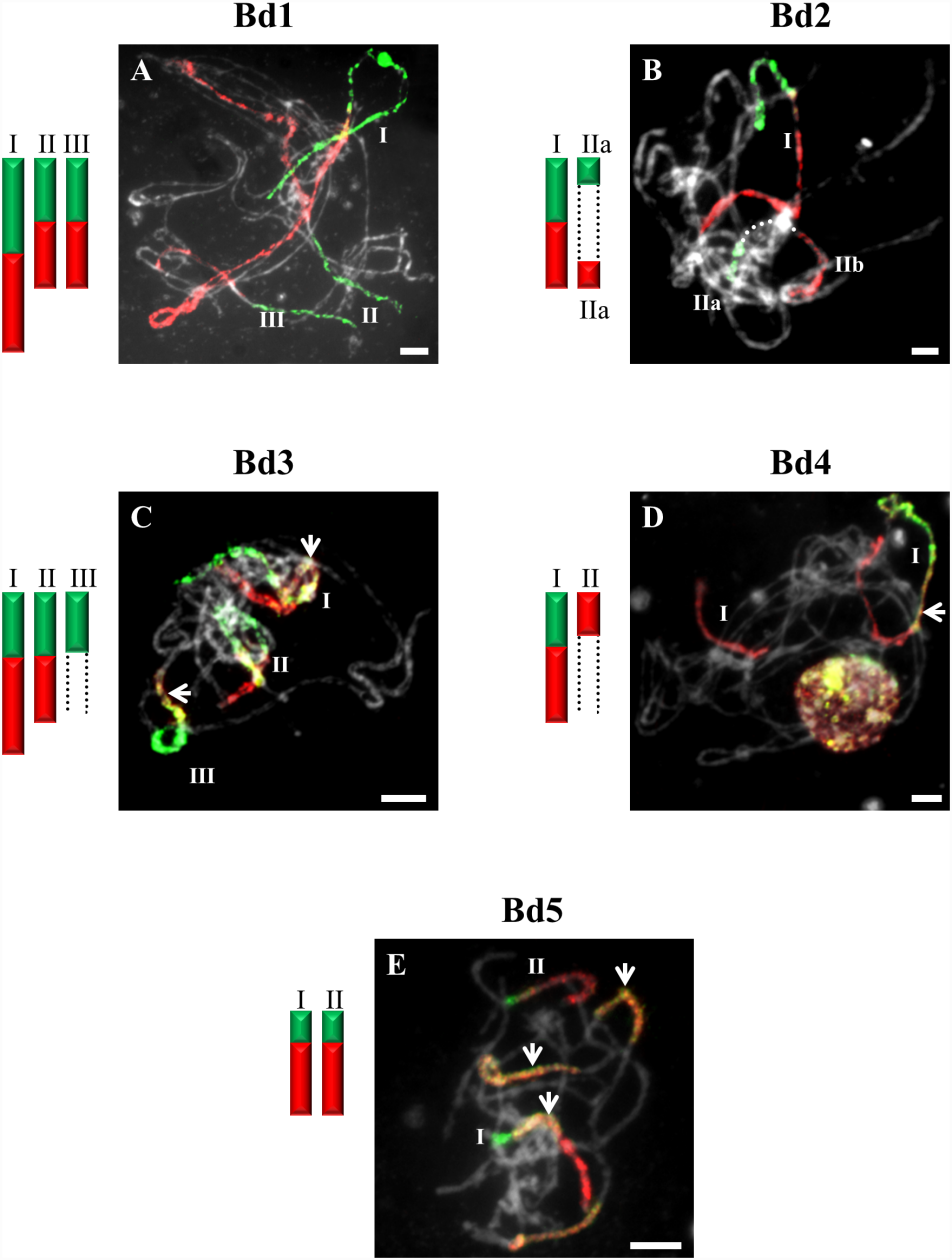
CCP in pachytene bivalents of *B. hybridum* (2n = 30) using BAC pools spanning the short (green fluorescence) and long (red fluorescence) arms of all five (Bd1–Bd5) *B. distachyon* chromosomes. Bd1- (**A**), Bd2- (**B**), Bd3- (**C**), Bd4- (**D**), Bd5-specific probes (**E**). All other information as in Figure 1. Bar: 10 µm.

The probes comprising BAC clones specific for Bd1 identified three bivalents (I–III) in pollen mother cells (PMCs) of *B. sylvaticum* (Figure 1A), *B. arbuscula* and *B. hybridum.* By contrast, only two bivalents are painted in *B. pinnatum* 2n = 16 (Figure 2A) and *B. stacei* (Figure 3A). The bivalents of *B. sylvaticum*, *B. arbuscula* and *B. pinnatum* 2n = 16 are the same size and hybridise with the Bd1-derived probes along their entire length (Figure 1A). By contrast, the labelled bivalents of *B. hybridum* differ in size (Figure 4A), one (designated I) being at least twice as long as the other two (II and III) resulting from the respective inherited chromosomes of progenitors *B. distachyon* and *B. stacei* in this allotetraploid. Due to problems with flower induction of *B. pinnatum* 2n = 18 under greenhouse conditions, the BAC clones from Bd1 were hybridised to somatic chromosomes only. However, as would be expected, three pairs of chromosomes (numbered I, II and III; Figure 2B) were painted, which resembles the pattern observed in all the other diploids in this study.

**Figure 5:**
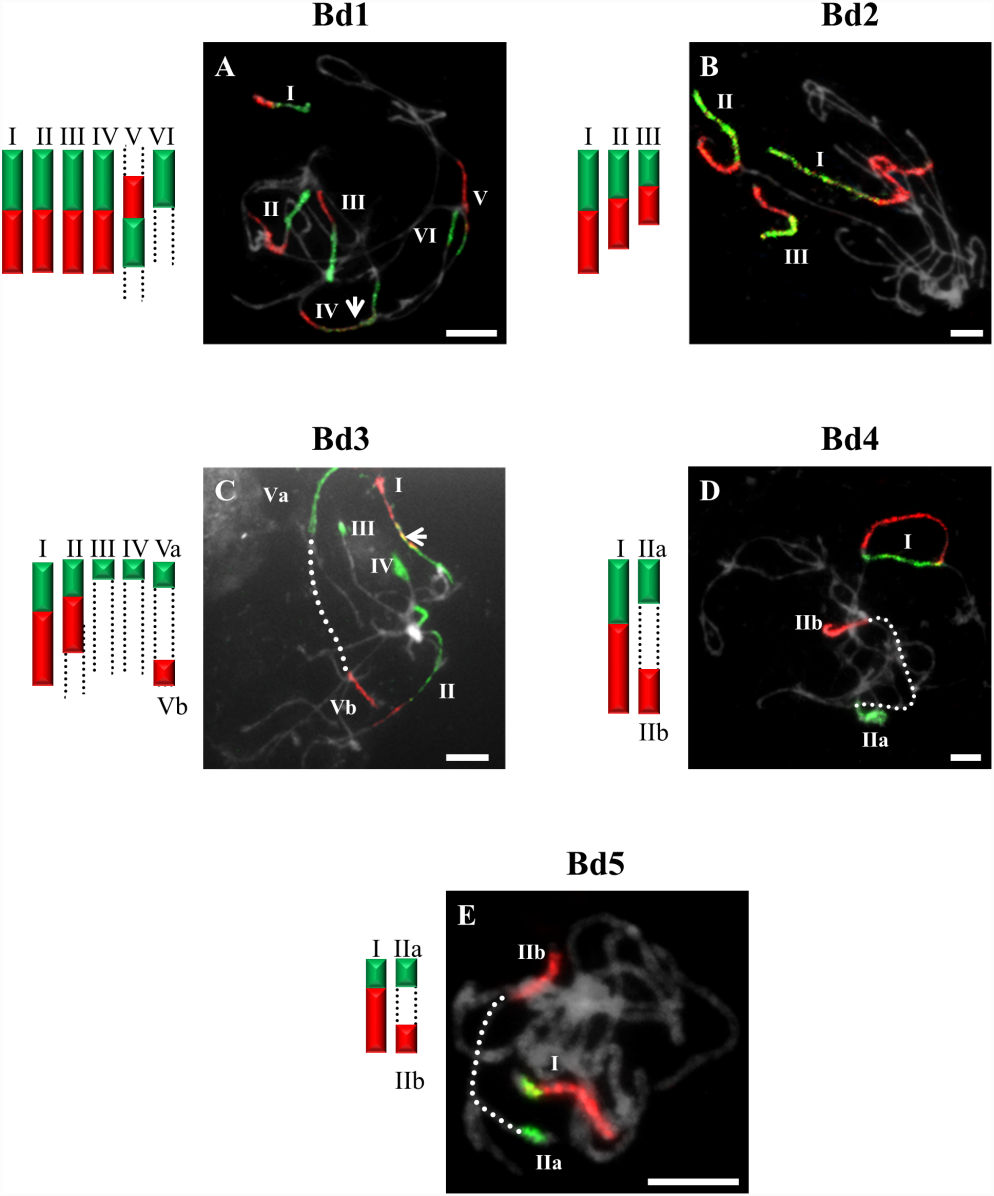
CCP in pachytene bivalents of *B. pinnatum* (2n = 28) using BAC pools spanning the short (green fluorescence) and long (red fluorescence) arms of all five (Bd1–Bd5) *B. distachyon* chromosomes. Bd1- (**A**), Bd2- (**B**), Bd3- (**C**), Bd4- (**D**), Bd5-specific probes (**E**). All other information as in Figure 1. Bar: 10 µm.

**Figure 6:**
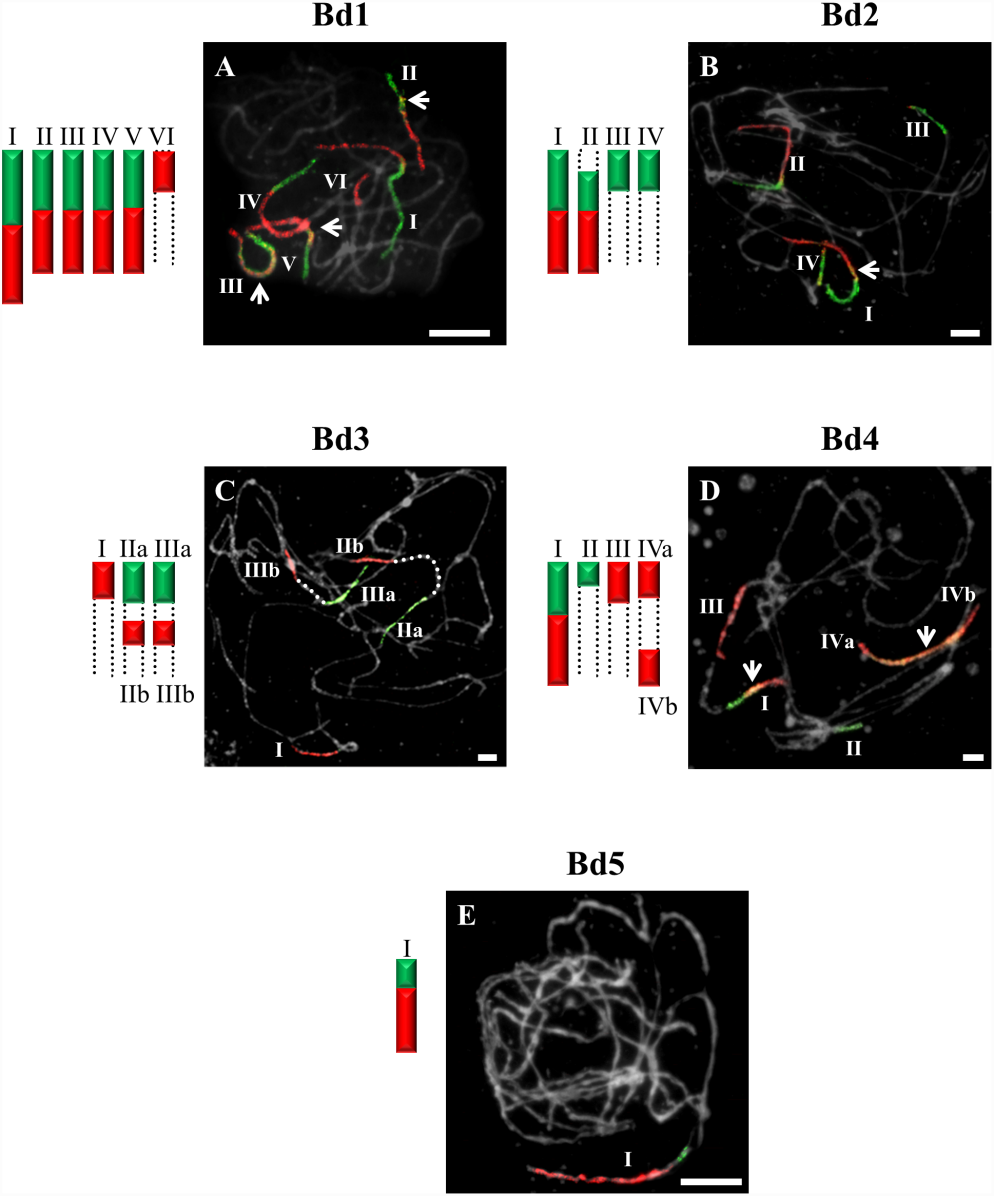
CCP in pachytene bivalents of *B. mexicanum* (2n = 40) using BAC pools spanning the short (green fluorescence) and long (red fluorescence) arms of all five (Bd1–Bd5) *B. distachyon* chromosomes. Bd1- (**A**), Bd2- (**B**), Bd3- (**C**), Bd4- (**D**), Bd5-specific probes (**E**). All other information as in Figure 1. Bar: 10 µm.

**Figure 7:**
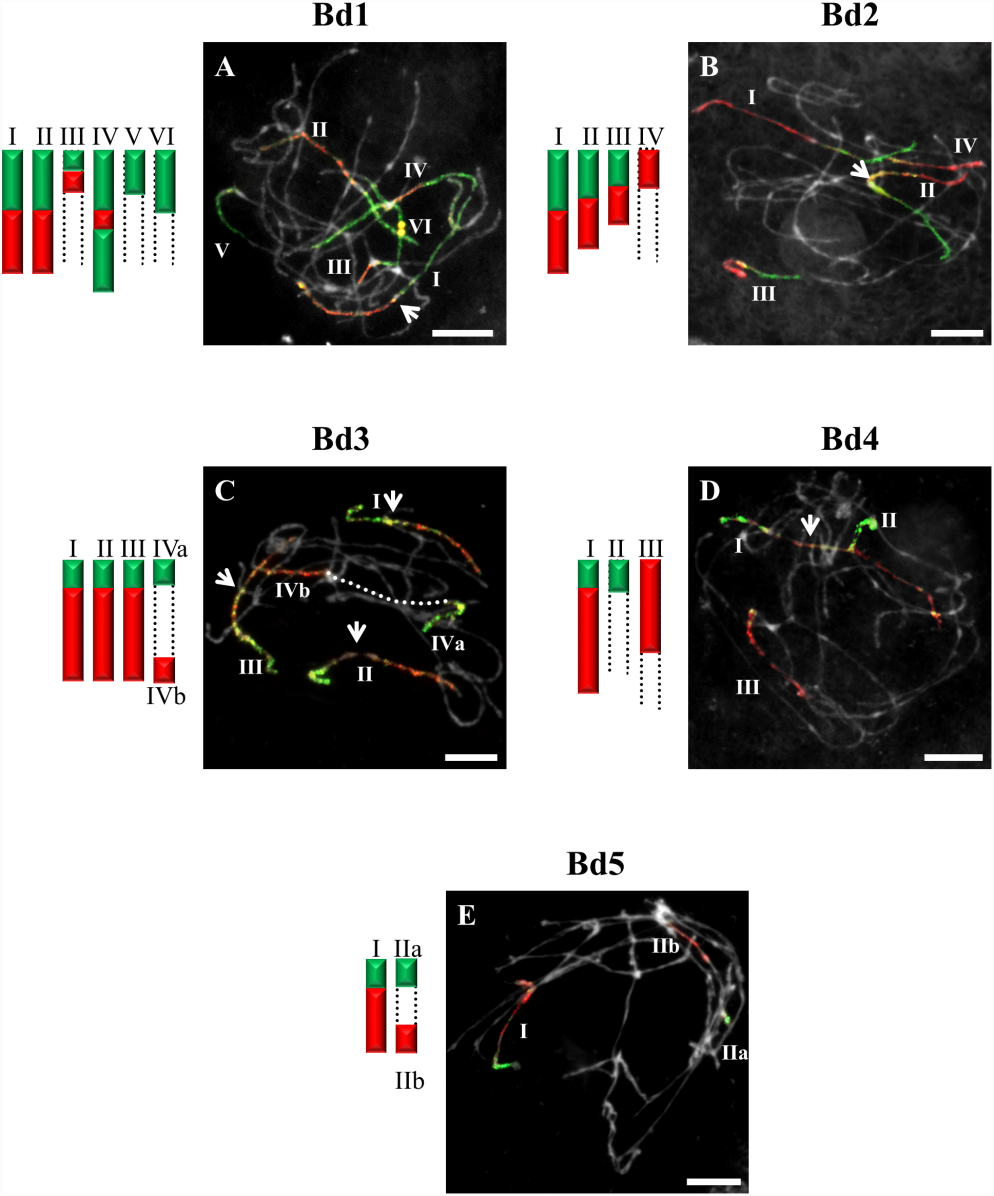
CCP in pachytene bivalents of *B. retusum* (2n = 38) using BAC pools spanning the short (green fluorescence) and long (red fluorescence) arms of all five (Bd1–Bd5) *B. distachyon* chromosomes. Bd1- (**A**), Bd2- (**B**), Bd3- (**C**), Bd4- (**D**), Bd5-specific probes (**E**). All other information as in Figure 1. Bar: 10 µm.

CCP with probes specific for Bd1 highlighted six bivalents in *B. pinnatum* 2n = 28 (Figure 5A), *B. phoenicoides*, *B. mexicanum* (Figure 6A) and *B. retusum* (Figure 7A). The bivalents numbered I–IV in *B. pinnatum* 2n = 28 and in *B. phoenicoides* have signals along their entire lengths (Figure 5A), whereas the bivalent designated V in *B. pinnatum* 2n = 28 and *B. phoenicoides* has signals only in the middle part of the chromosomes, the distal regions remaining unpainted (Figure 5A). The bivalents designated I–V in *B. mexicanum* (Figure 6A) and I–III in *B. retusum* (Figure 7A) were painted by Bd1S and Bd1L. However, it should be noted that the painted segment of Bd1 on bivalent III in *B. retusum* does not cover the whole length of the chromosome. The bivalent designated I in *B. mexicanum* is longer than the other four (Figure 6A), whilst the bivalent numbered IV in *B. retusum* contains two segments of Bd1S separated by a segment of Bd1L (Figure 7A). Additional bivalents designated VI in *B. pinnatum* 2n = 28 (Figure 5A) and *B. phoenicoides* and V–VI in *B. retusum* (Figure 7A) contain Bd1S segments, and bivalent VI in *B. mexicanum* (Figure 6A) contains a Bd1L segment.

### Bd2-derived pools of clones

CCP with BAC clones of the short and long arms of Bd2 revealed a strong signal along the entire length of bivalents designated I in *B. stacei* (Figure 3B), *B. hybridum* (Figure 4B) and in *B. mexicanum* (Figure 6B), I and II in *B. sylvaticum* (Figure 1B), and bivalents I–III in *B. pinnatum* 2n = 28 (Figure 5B), *B. phoenicoides* and *B. retusum* (Figure 7B). The bivalents I, II and III in *B. phoenicoides* were similar in size, unlike those of *B. pinnatum* 2n = 28 (Figure 5B) and *B. retusum* (Figure 7B) which varied in size. The bivalent designated II in *B. mexicanum* (Figure 6B) has Bd2S and Bd2L signals but they do not cover the whole length of the chromosomes. It should be noted that probes specific for Bd2S and Bd2L highlighted additional segments designated IIa and IIb of the same bivalent of *B. hybridum,* but there is an intervening unlabelled segment (Figure 4B). Furthermore, single segments of Bd2S designated II in *B. stacei* (Figure 3B) and III and IV in *B. mexicanum* (Figure 6B), and single segments of Bd2L numbered IV in *B. retusum* (Figure 7B) clearly do not span the entire length of the bivalents.

### Bd3-derived pools of clones

Only one bivalent designated I of *B. sylvaticum* (Figure 1C) and *B. pinnatum* 2n = 16 and 2n = 18 contains both short and long arm labels. Interestingly, a discontinuity of the hybridisation signal between the labelled short (Ia) and long arm (Ib) was observed in *B. arbuscula* (Figure 2C). Segments of Bd3S called I in *B. stacei* (Figure 3C), II in *B. sylvaticum* (Figure 1C), *B. pinnatum* 2n = 16, 2n-18 and in *B. arbuscula,* and Bd3L segments designated II in *B. stacei* (Figure 3C) are painted by the clones from Bd3S and Bd3L, respectively.

The bivalents called I and II in *B. pinnatum* 2n = 28 (Figure 5C), *B. phoenicoides* and *B. hybridum* (Figure 4C) hybridise with both the short and long arm probes of Bd3. They are both painted along their entire lengths in *B. hybridum* (Figure 4C), whereas bivalent I is painted completely but bivalent II only partially in *B. pinnatum* 2n = 28 and *B. phoenicoides* (Figure 5C). The bivalents called III in *B. hybridum* (Figure 4C) and III–IV in *B. pinnatum* 2n = 28 (Figure 5C) and *B. phoenicoides* have only the Bd3S segment. In addition, these two species contained a bivalent with terminal Bd3S- and Bd3L-derived segments (designated Va and Vb, respectively).

Bd3-specific probes highlight three bivalents of *B. mexicanum* (Figure 6C). The bivalent designated I hybridises only with the Bd3L probe so does not cover its entire length. Two other bivalents (IIa-b and IIIa-b) have terminal and interstitial segments separated by unpainted regions. The Bd3-specific probes hybridise to four bivalents of *B. retusum* (Figure 7C), but there is a discontinuity of signal in bivalent IVa–IVb.

### Bd4-derived pools of clones

One bivalent is highlighted completely with Bd4-derived probes in *B. pinnatum* 2n = 16, 2n = 18, *B. arbuscula* and *B. sylvaticum* (Figure 1D). The same set of probes hybridises with two bivalents of *B. pinnatum* 2n = 28 (Figure 5D), *B. phoenicoides* and *B. hybridum* (Figure 4D) and three bivalents of *B. retusum* (Figure 7D). Only bivalent I in all allotetraploids investigated and in *B. retusum* hybridises with the Bd4 probes along its entire length. The segments of Bd4S/L designated as IIa and IIb in *B. pinnatum* 2n = 28 and *B. phoenicoides* (Figure 5D) are localised at the two distal ends of the same bivalent. A Bd4L-specific segment occupies the distal part of the bivalent designated II in *B. hybridum* (Figure 4D) and III in *B. retusum* (Figure 7E). Additionally, a distal *B. retusum* segment hybridises to Bd4S-derived probes in bivalent numbered II. The Bd4S- and Bd4L-specific probes paint one arm only of two separate bivalents (I and II) of *B. stacei* (Figure 3D).

CCP of the BAC pools from Bd4 highlights four bivalents in *B. mexicanum* (Figure 6D). Bivalent I hybridises with the Bd4S- and Bd4L-specific probes along its entire length. The distal segments of bivalents II and III hybridise with either Bd4S or Bd4L. Two Bd4L-positive segments, designated IVa and IVb localise to both distal regions of another bivalent.

### Bd5-derived pools of clones

The Bd5-specific probes hybridise with only one bivalent in all diploids, i.e. *B. sylvaticum* (Figure 1E), *B. pinnatum* 2n = 16 and 2n = 18, *B. arbuscula*, *B. stacei* (Figure 3E) as well as in *B. mexicanum* (Figure 6E). The Bd5S and Bd5L probes paint both arms completely in all species except *B. arbuscula* where there is a discontinuity between the Bd5S-positive (Ia) and Bd5L-positive (Ib) segments (Figure 2D).

The Bd5-specific probes highlight the entire length of one bivalent of the allotetraploids *B. pinnatum* 2n = 28 (Figure 5E) and *B. phoenicoides,* and *B. retusum* (Figure 7E), and two bivalents of *B. hybridum* (Figure 4E). Additional Bd5S- (IIa) and Bd5L-positive (IIb) segments separated by an unpainted fragment are found in *B. pinnatum* 2n = 28 (Figure 5E), *B. phoenicoides* and *B. retusum* (Figure 7E).

## Discussion

### Genome evolution in *Brachypodium* diploids

Evolution amongst species of the model genus *Brachypodium* is of value in unravelling the biological processes involved in the origin of extant diploid and polyploid angiosperms. Recent advances in the genomics and phylogenetics of *Brachypodium* have been widely discussed [17], [19], [20], [43]. The nuclear genome of *B. distachyon* has been sequenced and the evolution of its karyotype has been attributed to seven major chromosome fusions from the putative 12-chromosomes intermediate grass ancestor. Indeed, detailed sequence analysis of the *B. distachyon* genome has revealed footprints of centromeric repeats and abundance of retrotransposon elements at the junctions of ancestral chromosome insertions [19], [44].

Using extensive comparative genomics analysis, the International Brachypodium Initiative [19] and Salse [4] postulated that the five chromosomes of *B. distachyon* could have originated from a 12-chromosome intermediate ancestral grass genome by seven centric fissions and 14 centric fusions. According to this hypothesis, *B. distachyon* chromosomes Bd1, Bd3 and Bd4 were derived from two nested insertions of six entire ancestral chromosomes into the centromeric regions of, respectively, three chromosomes, whilst Bd2 was derived from one similar insertion of one entire ancestral chromosome into another one. In contrast to the others, Bd5 remained virtually unchanged from its putative ancestral chromosome. Experimental evidence in other grass species for the hypothesis initially proposed by Salse et al. [45] in crop grass species was further extended for the Triticeae by Luo et al. [46], who proposed the mechanisms responsible for the reduction of the basic chromosome number from 12 to 7. Such a reorganisation in karyotype structure was attributed not only to end-to-end chromosome fusions or translocations, but also by the insertions of four entire chromosomes into break points in the centromeric regions of four other chromosomes, with an additional fusion and minor translocation events. The first experimental evidence in support of this hypothesis within *Brachypodium* was recently presented by Idziak et al. [20] using barcoding of somatic Bd2 and Bd3 chromosomes. These authors concluded that dysploidy events, as well as translocations and duplications, played an important role in the evolution of the Bd2 and Bd3-like chromosomes in the karyotypes of four diploid and allotetraploid species of *Brachypodium*.

In our study, BAC clones derived from Bd1 highlighted three bivalents in all diploids with 2n = 18 (Figure 1A). The one fewer painted bivalents in *B. pinnatum* 2n = 16 compared to *B. pinnatum* 2n = 18 is likely to be the result of an unidentified chromosome rearrangement that occurred in the former accession. CCP with the Bd2-specific probes revealed two labelled bivalents in *B. sylvaticum* (Figure 1B), and hybridisation with clones from Bd3 and Bd4 showed only one labelled bivalent in this species. Additionally, small signals of Bd3S-linked probes were found, suggesting possible minor translocations or duplications of this chromosome region in another bivalent (Figure 1C). Only one bivalent was highlighted consistently by Bd5-derived BAC clones in all diploids of *Brachypodium* studied (Figures 1E and 3E). Our more extensive analysis involving S and L probes from the five chromosomes of the *B. distachyon* complement indicates, however, that the karyotype structure of the *Brachypodium* 2n = 18 species studied resembles more closely the hypothetical 12-chromosome intermediate ancestral genome than that of *B. distachyon*, as demonstrated by the hybridisation of BAC clones from Bd1, Bd2 and Bd5 to three, two and one bivalents of *Brachypodium* 2n = 18 species, respectively. Bd5-specific probes also paint a single bivalent in *B. stacei* (Figure 3E), whereas the remaining Bd1–Bd4 probes paint more than one bivalent but show different signals (Figure 3A–3D) compared with the other diploids. The observed dysploidy and different genomic rearrangements could be related to differences in divergence time between the more ancestral annual *B. stacei* and the other more recently evolved perennial diploids (x = 9) [43].

### Genome evolution in *Brachypodium* allotetraploids

**Figure 8:**
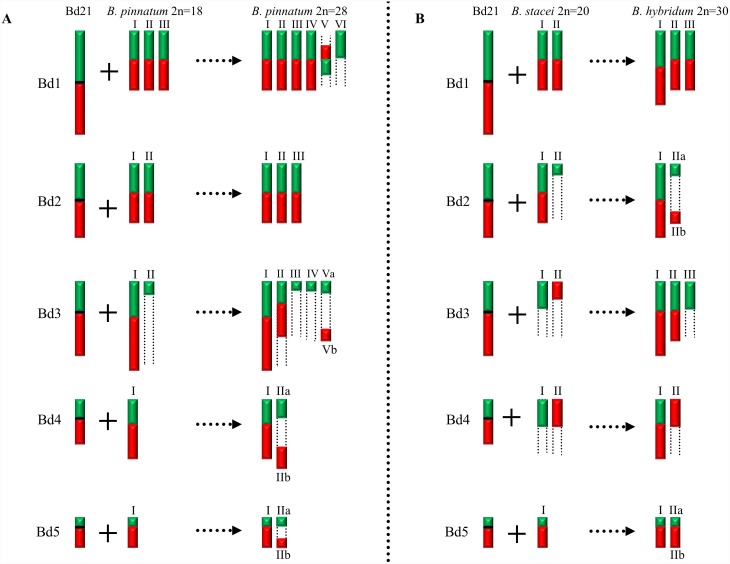
Diagrammatic summary of the patterns of CCP in pachytene bivalents of *B. distachyon* 2n = 10, *B. pinnatum* 2n = 16; 2n = 18, *B. pinnatum* 2n = 28 (A) and *B. distachyon* 2n = 10, *B. stacei* 2n = 20, *B. hybridum* 2n = 30 (B). Hybridisation with the probes derived from the short arm of the respective *B. distachyon* chromosome is consistently shown in green and from the long arm in red. Centromeres of *B. distachyon* chromosomes are represented by horizontal black bars. Relative lengths of chromosomes are only approximate.

Three allotetraploid species (*B. pinnatum* 2n = 28, *B. phoenicoides* 2n = 28 and *B. hybridum* 2n = 30) were analysed by CCP in this study. Allotetraploids *B. pinnatum* (Figure 5) and *B. phoenicoides* (data not shown) have a similar painting pattern in their genomes. The allotetraploid status of *B. pinnatum* 2n = 28, *B. phoenicoides* and *B. hybridum* was suggested by Wolny and Hasterok [41] using genomic *in situ* hybridisation (GISH). For many years, GISH was the method of choice for identifying putative ancestral species of various allopolyploids. However, if the repetitive sequences of the constituent genomes are too similar or if extensive homogenisation of these sequences between the parental species had occurred, the allopolyploid status cannot be confirmed by GISH [47]. Multicolour GISH in *B. pinnatum* 2n = 28 with total genomic DNA from *B. distachyon* and *B. pinnatum* 2n = 18 enabled the discrimination of ten and 18 chromosomes. The conclusion was that *B. pinnatum* 2n = 28 is in fact an interspecific hybrid and contains genomes that are identical or similar to the genomes of *B. distachyon* and *B. pinnatum* 2n = 18[41]. In the present study, the number of painted bivalents of Bd2, Bd4 and Bd5 in *B. pinnatum* 2n = 28 (Figure 5B, 5D–5E and Figure 8A) was equal to the sum of those in the *B. distachyon* and *B. pinnatum* 2n = 16; 2n = 18 genomes, confirming the putative allotetraploid nature of *B. pinnatum* 2n = 28 [41]. A similar conclusion was reached for *B. phoenicoides*, where the number of painted bivalents of Bd2, Bd4 and Bd5 was equal to the sum of those of *B. distachyon* and a second progenitor whose taxonomic identity remains unclear. Morphologically, *B. phoenicoides* is glabrous like *B. rupestre* 2n = 18, having mutique (non-awned) lemmas, twisted spikelets and partially inrolled leaves [42]. The second putative ancestor of *B. phoenicoides* was unresolved cytologically by GISH [41]. In addition, phylogenetic analyses of different genes did not clearly reveal the identity of the putative second ancestor of *B. phoenicoides* [17], [42], [43], [48], [49]. Bayesian phylogenetic trees based on multicopy ribosomal ITS genes and low-copy GIGANTEA genes revealed close relationships between *B. phoenicoides*, *B. pinnatum* and *B. sylvaticum* [17], [43], those based on multicopy ribosomal ETS showed a relationship between *B. phoenicoides* and *B. sylvaticum* only [43], whereas those based on the low-copy CAL gene showed close relationships between *B. phoenicoides, B. pinnatum* 2n = 18 and *B. rupestre* [17]. Further analysis using more informative sequence data and cytogenetic markers would be necessary to identify the putative second genome donor of *B. phoenicoides*, either extant or extinct.

GISH with genomic DNA from *B. distachyon* and *B. stacei* to the *B. hybridum* 2n = 30 genome discriminated 10 and 20 chromosomes, respectively [40]. CCP in *B. hybridum* corroborates the evidence for its hybrid origin from *B. stacei* and *B. distachyon*. The pattern of hybridisation of the Bd1-specific probes was the same as that of single BACs [36], with the number of painted chromosomes equalling the sum of the painted chromosomes in the two genome donors (Figure 4A and Figure 8B). Worthy of note is that one of the painted bivalents is longer than the others and is presumably derived from the larger *B. distachyon* genome, whilst the two shorter bivalents may have been derived from *B. stacei* (Figure 4A). The absence of a labelled distal segment may be with the result of chromosome restructuring, which is a frequent phenomenon in polyploid genomes [50]. The number of chromosomes painted by Bd5 is also equal to the sum of those painted in the *B. distachyon* and *B. stacei* genomes (Figure 4E and Figure 8B).

CCP of the Bd1- and Bd3-specific probes in *B. pinnatum* 2n = 28 (Figure 5A and 5C) and *B. phoenicoides* as well as the Bd2-Bd4-specific probes in *B. hybridum* (Figure 4B–4D) revealed that the number of painted bivalents in these allopolyploids is not a simple sum of the respective chromosomes originating from *B. distachyon* and a second ancestor (Figure 8A–8B), implying multiple chromosomal rearrangements during the evolution and divergence of these species. Frequent and rapid chromosomal rearrangements during speciation have been shown in many other allotetraploids of *Brassica* [51], *Tragopogon* [52] and *Lilium* [53].

### Karyotype evolution in the genus *Brachypodium*

CCP in *B. stacei* (Figure 3), *B. mexicanum* (Figure 6) and *B. retusum* (Figure 7) shows a distinctive pattern of hybridisation that is different from that observed for all the other species investigated. As mentioned above, Bd5 is the most conserved chromosome and has remained virtually unchanged through dysploidy events in *B. distachyon*. According to our results, only one bivalent is highlighted by the Bd5-specific probes in *B. stacei* (Figure 3E) and *B. mexicanum* (Figure 6E). Hasterok et al. were the first to suggest that *B. stacei* is a diploid species and that *B. distachyon* is unlikely to be the progenitor of *B. stacei* [40]. This hypothesis was later supported by phylogenetic analyses using both plastid (*ndh*F, *trn*LF) and nuclear (ITS, ETS) genes [43] which inferred that *B. stacei* and *B. mexicanum* represent basal species in the genus *Brachypodium*. The exact evolutionary status of *B. mexicanum* is a controversial issue. It was suggested by Catalan et al. [43] that it is either a tetra- or octoploid, implying that it should have more than one copy of the homoeologue for Bd5. Also, most perennial *Brachypodium* species have long, strong rhizomes, but the rhizomes of *B. mexicanum* are short. Furthermore, RAPD analysis was unable to resolve the phylogenetic position of *B. mexicanum* [48], [49]. The genomic polymorphism detected by RAPDs in *B. mexicanum* is probably connected with the geographical isolation of this taxon, which is probably a result of long-distance dispersal from a common ancestor widespread in a hypothetical ancestral Mediterranean-Eurasian area in the mid-late Miocene [43]. A phylogenetic reconstruction of the genus *Brachypodium* from combined sequences of a chloroplast *ndh*F gene and nuclear ITS showed both the presence of short 5S rDNA repeats, which are common for *B. distachyon* and *B. mexicanum*, and long 5S rDNA units, which are typical for all of the other species of *Brachypodium* [42]. Wolny et al. [17] concluded from a combined cytogenetic, CAL, GI and STT3-based phylogenetic analysis that an unidentified ancestral *Brachypodium* genome could be present in the modern *B. mexicanum*, *B. retusum*, *B. stacei* and *B. distachyon* genomes.

The Bd5 probe hybridised with two bivalents in *B. retusum* (Figure 7E). Wolny and Hasterok [41] hypothesed about the allopolyploid nature of *B. retusum* and suggested that *B. distachyon* could be one of its progenitors, with the identity of the other parent being unclear. They showed by GISH with genomic DNA from *B. pinnatum* 2n = 28 and *B. phoenicoides* the discrimination of 10 and 12 chromosomes, respectively. From the perspective of phylogenetic analysis, the position of *B. retusum* is still under debate [17], [42], [43]. A phylogeny of *Brachypodium* based upon combined *ndh*F and ITS data showed the nesting of *B. retusum* within the core-perennial clade, between the early diverging *B. arbuscula* and the most recent split of the core members of this clade (*B. pinnatum*, *B. rupestre*, *B. phoenicoides*, *B. sylvaticum*) [42]. However, the phylogenetic analysis based on CAL, DGAT and GI low-copy genes each detected two different copies in *B. retusum*, one in a basal or sub-basal position in the respective trees, sister to either the southern Spain endemic *B. boissieri* (DGAT, GI) or to *B. mexicanum* (CAL), and the other nested within the most recently evolved core perennial clade (*B. pinnatum*, *B. rupestre*, *B. phoenicoides*, *B. sylvaticum*) [43]. The latter results point to the existence of paralogues in *B. retusum* [43] and hence the potential allopolyploid nature of this species. The putative allopolyploid *B. retusum* could have been derived from hybridization and genome doubling of at least one ancestral genome and one core-perennial genome.

The number and chromosomal localisation of 5S rDNA loci is usually a reliable indicator of ploidy level in the genus *Brachypodium* [41]. However, FISH with a 5S rDNA probe highlighted only one pair of these loci in *B. mexicanum* (data not shown) and *B. stacei* [40], which is the same number observed in *B. distachyon* [40]. In contrast, there are three pairs of 5S rDNA loci in *B. retusum* [41], which has about the same chromosome number as *B. mexicanum*. Furthermore, *B. retusum* shares a short 0.2 kb 5S rDNA family with the *B. distachyon*/*B. mexicanum* group, and a long, 0.3 kb 5S rDNA family with other representatives of the genus [54].

### Conclusions

**Figure 9:**
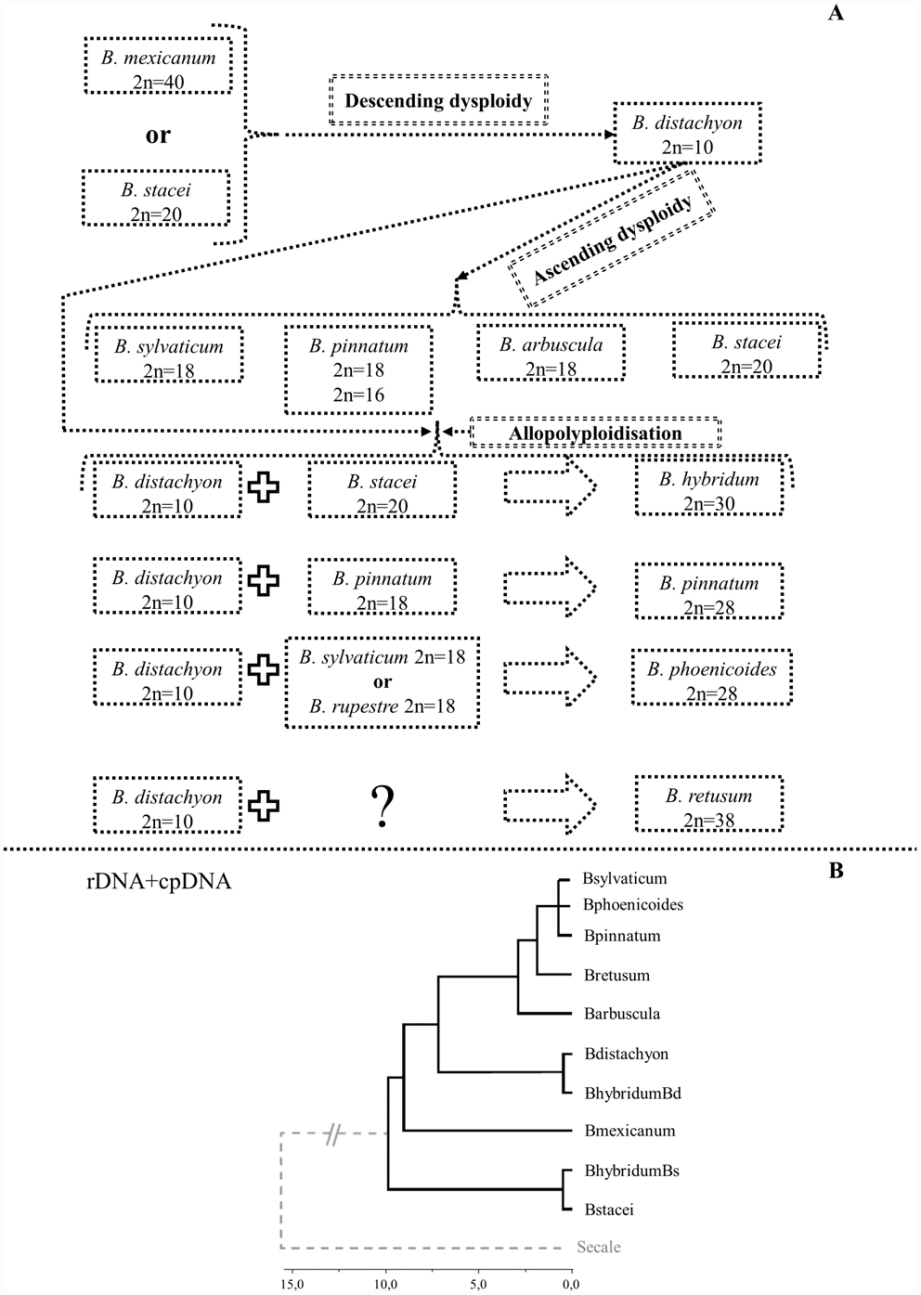
Model of karyotype evolution in the genus *Brachypodium* inferred from species containing a putative ancestral *Brachypodium* genome (*B. mexicanum* or *B. stacei*). According to the model, *B. distachyon* 2n = 10 is the intermediate species between *B. mexicanum* or *B. stacei* (**A**). Bayesian phylogenetic tree of *Brachypodium* representatives and an outgroup species showing relationships within the genus *Brachypodium* (**B**). The tree contains combined data from the cpDNA and rDNA analyses. Chronogram taken from [43] and modified by drop.tip (R ape package).

On the basis of published data [17], [41]–[43], [48], [49] and the results of this research, we propose two alternative hypothetical models of karyotype evolution in *Brachypodium*. According to the first model, the *B. distachyon* genome is formed after several rounds of descending dysploidy (for example by chromosome fusions) from one of the species containing a putative ancestral *Brachypodium* genome (*B. mexicanum* or *B. stacei*) (Figure 9A). According to this model, all of the diploids investigated, i.e. *B. sylvaticum*, *B. pinnatum* 2n = 16; 2n = 18, and *B. arbuscula* evolved from the *B. distachyon* genome via ascending dysploidy. The allotetraploids, such as *B. pinnatum*, *B. phoenicoides, B. hybridum* and *B. retusum* are the product of interspecific hybridisation events between some of the diploid species and *B. distachyon*. The results of our study suggest that dysploidy may play an important role in the evolution of different *Brachypodium* species, in a similar way to the evolution of species of Brassicaceae [28]. Such dysploidy-related fission or fusion events, though potentially responsible for rapid and significant changes in chromosome numbers, do not entail major changes in DNA content. Noticeably, this hypothesis is in accordance with recent phylogenetic analyses of Catalan et al. [43]. The Bayesian tree (Figure 9B) based on rDNA and cpDNA sequences shows the basal position of *B. stacei* and *B. mexicanum* with respect to other representatives in the genus *Brachypodium*. Furthermore, a sister relationship of *B. distachyon* with core perennial clade was shown.

**Figure 10:**
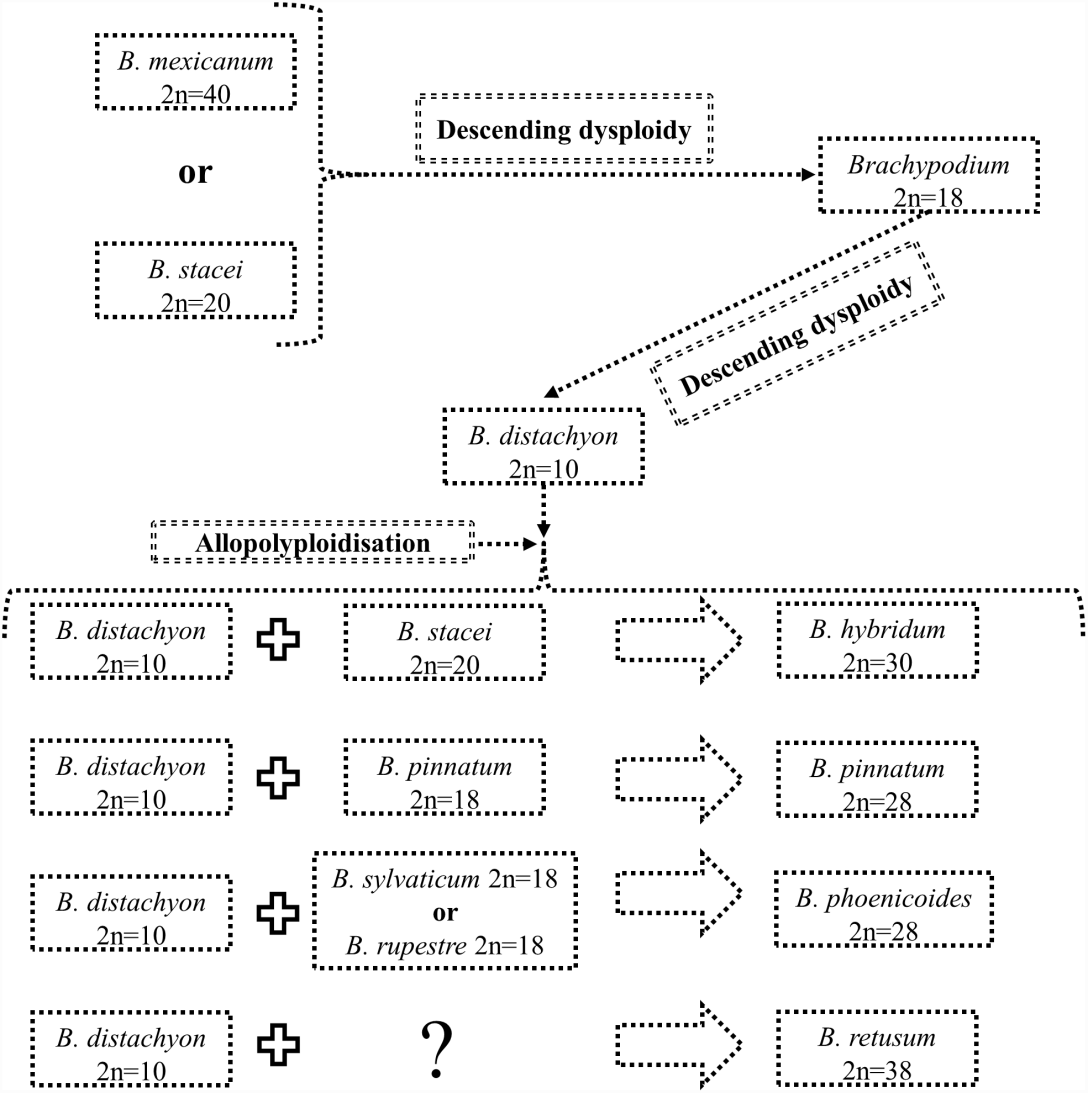
Model of karyotype evolution in the genus *Brachypodium* from *B. mexicanum* or *B. stacei*, describing a putative ancestral *Brachypodium* genome and proposing *Brachypodium* 2n = 18 as intermediate species between *B. mexicanum* 2n = 40 or *B. stacei* 2n = 20 and *B. distachyon* 2n = 10.

According to the second model, the *B. distachyon* genome was also formed from *B. mexicanum* or *B. stacei* via descending dysploidy, but with a *Brachypodium* 2n = 18-like genome as an intermediate (Figure 10). Clones derived from Bd1, Bd2 and Bd5 are present in the chromosomes of *Brachypodium* 2n = 18, as was proposed for the 12-chromosome intermediate grass ancestor [4], [19].

It would be worthwhile to extend the cytomolecular analyses of grass karyotype evolution to other members of the Poaceae, using the research infrastructure and resources developed for *B. distachyon*. Furthermore, in future experiments it would be of interest to design painting probes according to rice-*B. distachyon* collinearity patterns [19]. Some promising cross-genus BAC-FISH experiments mapping *B. distachyon* BAC clones to *Hordeum vulgare* have been reported [55], in which the authors demonstrate synteny at the chromosomal level between Bd1 of *B. distachyon* and *H. vulgare* chromosomes 2 H and 7 H. However, apart from this experiment, no other attempts to use BAC clones from *B. distachyon* to map other grass genera have been reported. It has to be assumed, therefore, that the ubiquitous repetitive DNA of plant genomes is thwarting similar CCP-based analyses.

## Materials and Methods

### Plant material and its origin

Nine species of *Brachypodium* were analysed. Seeds were obtained from various research centres and botanical gardens, details of which are given in Table 1. The plants to provide meiotic material were sown in pots filled with soil mixed with vermiculite at a ratio of 3∶1, and grown in a greenhouse under 16 h days at 20±1°C, illuminated by lamps emitting white light at an intensity of 10,000 lx. In order to induce synchronised flowering, 4-week-old plants of all species except *B. arbuscula* and *B. mexicanum* were vernalised for six weeks at 4°C. Immature spikes of *B. phoenicoides* 2n = 28 and *B. retusum* 2n = 38 were collected from the wild (Table 1) from locations where no specific permissions were required. As far as we are aware, our study did not involve endangered or protected species.

**Table 1:** Identities, somatic chromosome numbers (2n), localities and sources of the *Brachypodium* material used in this study.

Species	Accessionnumber	2n	Locality	Source*
*B. arbuscula*	n/a	18	Spain: Canaryisles, Gomera	*a*
*B. distachyon*	Bd21(PI 254867)	10	Iraq(genome sequenced)	*b*
*B. hybridum*	ABR113	30	Portugal: Lisboa	*c*
*B. phoenicoides*	n/a	28	Spain: Huesca(42° 07′ 11.05″ N 0° 26′ 18.57″ W)	*d*
*B. pinnatum*	PI 185135	16	Iran	*b*
	PI 345982	18	Norway	*b*
	n/a	28	the Netherlands:Scherpenzeel	*e*
			(52° 04′ 41.51″N 5° 28′ 34.65″E)	
*B. retusum*	n/a	38	Spain: Huesca(42° 09′ 08.96″ N 0° 20′ 41.23″ W)	*d*
*B. mexicanum*	Bmex347	40	Mexico: Hidalgo,Sierra de Pachuca	*c*
*B. stacei*	ABR114	20	Spain: Balearicisles, Formentera	*c*
*B. sylvaticum*	PI 297868	18	Australia	*c*

*a High Polytechnic School of Huesca, University of Zaragoza, Huesca, Spain; *b* US Department of Agriculture–National Plant Germplasm System, USA; *c* Institute of Biological, Environmental and Rural Sciences, Aberystwyth University, UK; *d* Collected from the wild by Alexander Betekhtin; *e* Private collection of Dr Ger Londo, Scherpenzeel, the Netherlands.

### Preparation of meiotic and mitotic chromosome squashes

Preparation of meiotic chromosome squashes followed published methodology [35], [37]. Briefly, individual anthers were isolated using fine needles in a watch glass with a 10 mM citrate buffer then digested enzymatically for 2 h at 37°C in a mixture comprising 10% pectinase (Sigma, cat. no. P-0690), 0.65%, cellulase “Onozuka R-10” (Serva, cat. no. 16419.02), 0.5% cellulase (Calbiochem, cat. no. 21 947), 0.15% cytohelicase (Sigma, cat. no. C-8274) and 0.15% pectolyase (Sigma, cat. no. P-3026). The anthers were transferred to a slide in a drop of 45% acetic acid, covered with a coverslip, gently squashed and frozen on dry ice. The coverslips were flicked off with a blade and the slides were air-dried.

For *B. pinnatum* PI 345982, mitotic chromosome preparations were made using the methodology described in [37], [38]. In brief, enzymatic digestion of roots was carried out for 2 h at 37°C in a mixture comprising 20% pectinase (Sigma, cat. no. P-0690), 1% cellulase (Calbiochem, cat. no. 21 947) and 1% cellulase “Onozuka R-10” (Serva, cat. no. 16419.02) in 10 mM citrate buffer. The meristems were extruded in 45% acetic acid and transferred to a slide, covered with a coverslip and squashed. Further steps in the procedure were same as for meiotic chromosome preparations.

### Probes for chromosome painting and FISH

The BAC clones used for chromosome painting of *Brachypodium* species came from the BD_ABa and BD_CBa genomic DNA libraries, and were selected from the five assemblies of FingerPrinted Contigs previously aligned to the *B. distachyon* karyotype [18]. In order to minimise the risk of cross-hybridisation, clones from centromeric and pericentromeric regions and (with a few exceptions) those containing more than 30% of repeats were excluded from the painting pools. The characteristics of BACs spanning respective *B. distachyon* chromosome arms are shown in S1–S5 Tables.

BAC DNA was isolated using a standard alkaline lysis method followed by labelling with custom-made [56] digoxigenin-dUTP for short chromosome arms and Cy3-dUTP for long chromosome arms using nick translation as described in [57]. Detailed lists of BAC clones comprising the pools for individual chromosome arms can be requested from the authors. Fluorescence *in situ* hybridisation (FISH) was based on the protocol published in Idziak et al. [35] and Jenkins and Hasterok [37] with minor modifications. Pooled BAC DNA was precipitated and dissolved in a hybridisation mixture including 40% deionised formamide, 15% dextran sulphate and 2×SSC (saline sodium citrate). Hybridisation mixtures with probes were pre-denatured for 10 min at 75°C, then denatured together with substrate DNA on slides for 4.5 min at 73°C and allowed to hybridise in a humid chamber for about 48 h at 37°C. Post-hybridisation washes were performed in 10% formamide in 2×SSC for 2×5 min at 37°C (equivalent to 59% stringency). Digoxigenated probes were immunodetected using FITC-conjugated anti-digoxigenin antibodies (Roche, cat. no. 11 207 741 910) according to standard protocol, while the Cy3-dUTP probes were directly visualised. The preparations were mounted and counterstained in Vectashield (Vector Laboratories, cat. no. H-1000) containing 2.5 µg/ml of 4′,6-diamidino-2-phenylindole (DAPI; Sigma, cat. no. D-9564).

The preparations were analysed and photomicrographs were acquired using an Olympus wide-field Provis AX microscope with narrow band filters and equipped with a monochromatic CCD camera (Retiga 2000R; QImaging) and illumination system based on a 100 W mercury lamp. All images were artificially coloured using Wasabi (Hamamatsu Photonics) and then uniformly processed and superimposed using Photoshop CS3 (Adobe) software.

## Supporting Information

S1 Table **Characteristics of BAC clones used for the chromosome painting of** ***B. distachyon*** **chromosome 1 (Bd1).**
(DOCX)

S2 Table **Characteristics of BAC clones used for the chromosome painting of** ***B. distachyon*** **chromosome 2 (Bd2).**
(DOCX)

S3 Table **Characteristics of BAC clones used for the chromosome painting of** ***B. distachyon*** **chromosome 3 (Bd3).**
(DOCX)

S4 Table **Characteristics of BAC clones used for the chromosome painting of** ***B. distachyon*** **chromosome 4 (Bd4).**
(DOCX)

S5 Table **Characteristics of BAC clones used for the chromosome painting of** ***B. distachyon*** **chromosome 5 (Bd5).**
(DOCX)
