# Supplementary material for: Reconstructing the Evolution of Brachypodium Genomes Using Comparative Chromosome Painting
Source: PLoS One. 2014 Dec 10;9(12):e115108. doi: 10.1371/journal.pone.0115108 (PMC4262448; doi:10.1371/journal.pone.0115108)
Supplement: S1 Table — Characteristics of BAC clones used for the chromosome painting of B. distachyon chromosome 1 (Bd1). (DOCX) [file pone.0115108.s001.docx]

**Table S1.** Characteristics of BAC clones used for the chromosome painting of *B. distachyon* chromosome 1 (Bd1).

Short (S) arm

| Clone name | Start (bp) | End (bp) | Repeat content (%) |
| --- | --- | --- | --- |
| a0035K02 | 147863 | 304506 | 18.31 |
| b0027N17 | 560624 | 710332 | 6.56 |
| a0037D23 | 1171403 | 1328435 | 13.07 |
| a0012F06 | 1537097 | 1734409 | 7.59 |
| a0032E05 | 1907231 | 2063694 | 11.83 |
| a0008O14 | 2635548 | 2801693 | 8.30 |
| a0021B03 | 3028832 | 3173186 | 6.23 |
| a0004B12 | 3276891 | 3460444 | 5.68 |
| b0044D24 | 3878248 | 4004060 | 13.91 |
| b0003A11 | 4404030 | 4546882 | 30.82 |
| a0032K13 | 5048843 | 5206517 | 26.89 |
| a0017K22 | 5375697 | 5509098 | 17.71 |
| b0037O18 | 6122656 | 6272292 | 19.17 |
| a0022C04 | 6574012 | 6741405 | 30.61 |
| b0040G07 | 7221475 | 7389553 | 30.09 |
| b0013P15 | 7656602 | 7805960 | 29.21 |
| a0013O16 | 7926543 | 8055682 | 39.20 |
| b0030L10 | 8680898 | 8845282 | 10.03 |
| b0012L20 | 8850673 | 9007358 | 14.08 |
| a0015H06 | 9424591 | 9569999 | 14.80 |
| a0007G23 | 9965773 | 10101237 | 15.91 |
| a0032D10 | 10490542 | 10652198 | 11.18 |
| a0003J21 | 10927667 | 11073109 | 16.93 |
| a0023P13 | 11505702 | 11632287 | 22.48 |
| a0032F08 | 12115980 | 12241228 | 17.12 |
| a0024M10 | 12444261 | 12575731 | 23.81 |
| a0027D04 | 12706461 | 12847057 | 22.87 |
| b0019G20 | 13362834 | 13517753 | 21.02 |
| b0023C02 | 13999817 | 14137163 | 13.45 |
| b0001G04 | 14561692 | 14709543 | 15.53 |
| a0020A04 | 15092918 | 15238493 | 0.00 |
| b0023O18 | 15449374 | 15577903 | 0.00 |
| a0003N21 | 16107590 | 16251236 | 10.80 |
| a0002N01 | 16344468 | 16496573 | 11.12 |
| a0009N18 | 17150298 | 17335777 | 0.00 |
| a0014L23 | 17404191 | 17543242 | 7.86 |
| b0018P22 | 18190466 | 18326563 | 23.15 |
| a0017E13 | 18574112 | 18708723 | 0.00 |
| a0010I03 | 19198770 | 19342731 | 7.06 |
| a0007L04 | 19364297 | 19511432 | 8.67 |
| b0002O16 | 20013520 | 20160236 | 0.00 |
| a0024N14 | 20488400 | 20631457 | 19.18 |
| a0027K03 | 21168673 | 21307307 | 0.00 |
| a0010K04 | 21496092 | 21643627 | 19.83 |
| a0011I01 | 21907910 | 22040598 | 0.00 |
| a0018B03 | 22412015 | 22565632 | 32.71 |
| b0022H13 | 23114454 | 23242441 | 0.00 |
| a0023E14 | 23230575 | 23392276 | 21.52 |
| a0043B06 | 24028749 | 24191469 | 22.76 |
| a0042C21 | 24228323 | 24375228 | 16.48 |
| a0026E19 | 25017625 | 25161139 | 14.31 |
| a0046B12 | 25556278 | 25718695 | 17.60 |
| a0018O15 | 25727688 | 25878318 | 13.18 |
| b0028A06 | 26442023 | 26591576 | 16.02 |
| b0002C04 | 27060765 | 27214938 | 27.84 |
| a0006K13 | 27522409 | 27682274 | 23.09 |
| a0044I06 | 28135872 | 28292480 | 21.81 |
| a0043P17 | 28526824 | 28683718 | 21.98 |
| a0029A09 | 28940084 | 29079192 | 22.18 |
| a0032C01 | 29475135 | 29677346 | 31.42 |
| a0002G12 | 30075959 | 30187436 | 25.08 |
| a0007B20 | 30824142 | 30835981 | 8.00 |
| a0036J15 | 31222238 | 31387974 | 13.86 |
| a0037D16 | 31313973 | 31498115 | 24.11 |
| a0018G20 | 32094917 | 32274028 | 12.54 |
| b0024I19 | 32507293 | 32633286 | 0.00 |
| b0014O02 | 33010624 | 33123772 | 0.00 |
| a0024G16 | 33587038 | 33745554 | 0.00 |
| b0037O03 | 33832455 | 34023561 | 20.87 |
| a0004L01 | 34316249 | 34466638 | 15.53 |

Long (L) arm

| Clone name | Start (bp) | End (bp) | Repeat content (%) |
| --- | --- | --- | --- |
| b0011C11 | 38625084 | 38768816 | 25.98 |
| a0002G19 | 39219901 | 39352626 | 20.11 |
| a0030K01 | 39352642 | 39424325 | 13.60 |
| a0002I22 | 39952805 | 40102980 | 19.01 |
| b0023K21 | 40363127 | 40508554 | 24.17 |
| a0003G01 | 41070484 | 41199669 | 5.68 |
| a0017I18 | 41400831 | 41536057 | 10.35 |
| a0006H08 | 42291978 | 42445395 | 30.82 |
| a0034B17 | 42516220 | 42665540 | 26.89 |
| a0022M24 | 43211660 | 43355407 | 17.71 |
| b0004P09 | 43536825 | 43670757 | 0.00 |
| b0003O14 | 43968448 | 44100412 | 0.00 |
| a0046B04 | 44701450 | 44835069 | 28.04 |
| a0009H21 | 45130478 | 45275483 | 30.09 |
| b0019N18 | 45624834 | 45763826 | 29.21 |
| b0039A23 | 45904934 | 46085862 | 34.61 |
| b0025P22 | 46564769 | 46692954 | 10.03 |
| a0045K11 | 47017729 | 47159607 | 14.08 |
| a0018I01 | 47327837 | 47472575 | 14.80 |
| a0018A03 | 48153393 | 48351371 | 12.13 |
| b0044L08 | 48612347 | 48783561 | 9.01 |
| a0016L07 | 49131764 | 49282548 | 17.24 |
| b0042L08 | 49567141 | 49756854 | 22.48 |
| a0003G14 | 50139085 | 50274374 | 17.12 |
| a0007A17 | 50488742 | 50629310 | 23.81 |
| a0002G03 | 50987420 | 51131768 | 21.02 |
| a0002M19 | 51404954 | 51599184 | 13.45 |
| b0035K24 | 51720482 | 51914140 | 15.53 |
| a0046C24 | 52577876 | 52717406 | 12.29 |
| a0020C13 | 52998818 | 53110321 | 14.83 |
| a0011F10 | 53395079 | 53532078 | 10.55 |
| a0046G17 | 54082210 | 54253048 | 0.00 |
| b0047M09 | 54775761 | 54934862 | 11.12 |
| b0036M23 | 55099293 | 55274619 | 14.70 |
| a0011D03 | 55361479 | 55401129 | 7.86 |
| b0044C20 | 56154348 | 56285508 | 16.71 |
| b0013C18 | 56402015 | 56523891 | 0.00 |
| b0028P17 | 57093738 | 57225377 | 0.00 |
| a0022N20 | 57208701 | 57348499 | 11.96 |
| a0019B04 | 58011832 | 58155264 | 19.18 |
| b0003K21 | 58350464 | 58480351 | 15.82 |
| a0010A14 | 59503419 | 59676696 | 0.00 |
| a0008E12 | 60079495 | 60251020 | 17.16 |
| a0005H16 | 60258365 | 60413281 | 0.00 |
| b0037A14 | 61095306 | 61288697 | 19.17 |
| a0012H18 | 61475648 | 61619144 | 0.00 |
| b0003A21 | 61920716 | 62077069 | 0.00 |
| a0034M17 | 62501248 | 62642447 | 0.00 |
| a0045D19 | 63062019 | 63221983 | 14.31 |
| b0022G04 | 63557791 | 63711230 | 17.60 |
| a0013D23 | 64120769 | 64297730 | 16.02 |
| b0011I02 | 64559074 | 64702171 | 27.84 |
| a0003I14 | 65067565 | 65202176 | 23.09 |
| a0046P14 | 65376014 | 65522455 | 21.81 |
| a0009I15 | 65946210 | 66098108 | 21.98 |
| b0026H13 | 66197674 | 66346594 | 22.18 |
| b0030D22 | 67065313 | 67205367 | 25.08 |
| a0019B19 | 67392232 | 67529032 | 8.00 |
| b0003K24 | 67945518 | 68072820 | 13.86 |
| a0011O07 | 68533765 | 68686250 | 24.11 |
| a0043A05 | 68898017 | 69053532 | 12.54 |
| b0004O01 | 69023274 | 69164463 | 23.47 |
| b0039M08 | 69966292 | 70146601 | 29.34 |
| a0040G14 | 70435911 | 70578835 | 17.94 |
| b0017K19 | 71146553 | 71281318 | 0.00 |
| a0021F18 | 71455475 | 71597258 | 0.00 |
| a0041A08 | 72027767 | 72181888 | 6.97 |
| b0002N07 | 72465040 | 72619352 | 4.45 |
| a0005K09 | 72948475 | 73083942 | 4.87 |
| b0039K17 | 73601518 | 73740071 | 5.12 |
| a0033F06 | 74020535 | 74180685 | 4.77 |
| b0035K23 | 74475472 | 74659792 | 12.79 |
