# Supplementary material for: Reconstructing the Evolution of Brachypodium Genomes Using Comparative Chromosome Painting
Source: PLoS One. 2014 Dec 10;9(12):e115108. doi: 10.1371/journal.pone.0115108 (PMC4262448; doi:10.1371/journal.pone.0115108)
Supplement: S2 Table — Characteristics of BAC clones used for the chromosome painting of B. distachyon chromosome 2 (Bd2). (DOCX) [file pone.0115108.s002.docx]

**Table S2.** Characteristics of BAC clones used for the chromosome painting of *B. distachyon* chromosome 2 (Bd2).

Short (S) arm

| Clone name | Start (bp) | End (bp) | Repeat content (%) |
| --- | --- | --- | --- |
| a0038A01 | 1022 | 132144 | 4.73 |
| a0026H23 | 501743 | 631176 | 5.15 |
| b0039C09 | 1311448 | 1507438 | 6.21 |
| a0027K15 | 1864643 | 2004976 | 4.27 |
| b0035C01 | 2500100 | 2659222 | 4.28 |
| b0002F19 | 2858161 | 3010741 | 5.64 |
| a0028O04 | 3492740 | 3587755 | 10.20 |
| b0048M15 | 3999943 | 4170302 | 18.58 |
| a0002P22 | 4319740 | 4509765 | 7.60 |
| a0045F24 | 6004397 | 6146555 | 12.87 |
| a0047D12 | 17856422 | 17996794 | 27.25 |
| a0021H13 | 18176419 | 18323680 | 28.06 |
| a0026K14 | 19861012 | 20005795 | 15.73 |
| a0043C22 | 20005803 | 20143867 | 25.91 |
| b0006D07 | 20880418 | 21008785 | 17.52 |
| b0015N23 | 21980992 | 22118800 | 12.71 |
| b0038L02 | 22509927 | 22639901 | 23.44 |
| b0011O19 | 25393226 | 25510444 | 14.12 |
| a0047M10 | 7007047 | 7144308 | 8.94 |
| a0019E04 | 8843922 | 9006117 | 13.8 |
| a0012B07 | 9006125 | 9148678 | 6.31 |
| a0005E09 | 10380990 | 10507985 | 10.31 |
| b0048L18 | 12984227 | 13000547 | 8.85 |
| a0044D02 | 14006553 | 14195269 | 11.1 |
| a0017D02 | 15866689 | 16021967 | 17.9 |
| a0031J04 | 16021976 | 16162135 | 12.06 |

Long (L) arm

| Clone name | Start (bp) | End (bp) | Repeat content (%) |
| --- | --- | --- | --- |
| b0031J17 | 33543920 | 33664342 | 15.14 |
| a0014K11 | 34309867 | 34503922 | 8.53 |
| b0003D21 | 35507055 | 35522066 | 19.01 |
| b0007E06 | 36376507 | 36505573 | 18.82 |
| b0022I07 | 38509106 | 38646001 | 9.09 |
| a0024L09 | 38997728 | 39192842 | 8.39 |
| b0031K20 | 39779799 | 39931474 | 5.43 |
| b0016E24 | 39997753 | 40003453 | 1.65 |
| a0043N06 | 41508421 | 41714064 | 18.78 |
| a0008H07 | 42500887 | 42664133 | 14.08 |
| b0018H13 | 42943153 | 43001509 | 9.64 |
| a0029H05 | 43505258 | 43648404 | 13.83 |
| a0009N24 | 44005924 | 44173470 | 13.71 |
| b0041G17 | 44876290 | 45007631 | 13.75 |
| b0031I09 | 46500135 | 46639653 | 7.51 |
| b0040K17 | 47000159 | 47021312 | 4.78 |
| b0019P09 | 48369110 | 48504229 | 3.43 |
| b0041J21 | 49505774 | 49706051 | 9.11 |
| b0038L04 | 50005019 | 50143082 | 5.40 |
| b0012J01 | 51003290 | 51006240 | 0.00 |
| a0031O24 | 52001822 | 52162247 | 4.16 |
| a0036P06 | 52875522 | 53003468 | 5.61 |
| a0027O24 | 53007896 | 53168487 | 8.87 |
| b0040O17 | 53370233 | 53504052 | 4.56 |
| b0036G07 | 53816466 | 54010118 | 3.84 |
| b0027N08 | 54420021 | 54540398 | 9.29 |
| b0047O03 | 55698147 | 55846468 | 5.48 |
| a0038M22 | 56336703 | 56502216 | 4.16 |
| a0038G14 | 57002804 | 57148130 | 7.24 |
