# Supplementary material for: Reconstructing the Evolution of Brachypodium Genomes Using Comparative Chromosome Painting
Source: PLoS One. 2014 Dec 10;9(12):e115108. doi: 10.1371/journal.pone.0115108 (PMC4262448; doi:10.1371/journal.pone.0115108)
Supplement: S3 Table — Characteristics of BAC clones used for the chromosome painting of B. distachyon chromosome 3 (Bd3). (DOCX) [file pone.0115108.s003.docx]

**Table S3.** Characteristics of BAC clones used for the chromosome painting of *B. distachyon* chromosome 3 (Bd3).

Short (S) arm

| Clone name | Start (bp) | End (bp) | Repeat content (%) |
| --- | --- | --- | --- |
| a0019P17 | 353866 | 501536 | 12.96 |
| a0001O14 | 501589 | 646912 | 10.33 |
| b0028O16 | 856255 | 1007650 | 12.86 |
| a0005B05 | 1000513 | 1146984 | 5.57 |
| a0011O22 | 1330674 | 1507458 | 7.88 |
| a0024P19 | 1507465 | 1643914 | 14.28 |
| b0026H03 | 1872780 | 2007217 | 6.12 |
| b0040M13 | 2318183 | 2506944 | 9.33 |
| b0022F20 | 2505550 | 2655293 | 9.03 |
| a0015A18 | 4001904 | 4157452 | 8.62 |
| a0007G18 | 4853075 | 4999533 | 15.21 |
| b0041N16 | 5017958 | 5207531 | 12.89 |
| a0015J14 | 5367097 | 5508104 | 25.79 |
| a0023I13 | 5506573 | 5699212 | 23.00 |
| a0018B12 | 6003300 | 6153924 | 11.42 |
| a0044L10 | 6362069 | 6506724 | 15.86 |
| a0001N07 | 6854023 | 7004002 | 7.62 |
| a0029A17 | 7006740 | 7159041 | 6.88 |
| a0042G11 | 7401273 | 7506116 | 18.09 |
| b0038O09 | 8001902 | 8135334 | 16.54 |
| a0030J22 | 8504730 | 8651070 | 13.91 |
| b0017F24 | 8911272 | 9006737 | 19.56 |
| b0047F12 | 9867071 | 10004537 | 28.22 |
| b0014A10 | 11356967 | 11507812 | 25.08 |
| b0016A22 | 11505050 | 11712720 | 21.97 |
| a0027M12 | 12326614 | 12559176 | 21.97 |
| a0006L03 | 12491194 | 12650638 | 26.19 |
| b0035L10 | 12501987 | 12660276 | 27.19 |
| b0023K19 | 12858741 | 13009244 | 21.57 |
| a0024A09 | 13372987 | 13528955 | 29.01 |
| b0010J18 | 13993335 | 14131952 | 16.02 |
| a0003B19 | 14467636 | 14508862 | 18.68 |
| a0014K09 | 14869360 | 15008860 | 15.03 |
| b0041F02 | 15002696 | 15181874 | 5.37 |
| a0022G01 | 16038657 | 16055486 | 5.49 |
| a0017B02 | 19002671 | 19154844 | 11.10 |
| b0009N11 | 19496003 | 19508052 | 2.37 |
| a0026C10 | 19503371 | 19692680 | 16.69 |
| a0019E11 | 19840599 | 19994588 | 21.87 |
| b0014A01 | 20363699 | 20508591 | 19.13 |
| b0001I10 | 21502253 | 21650260 | 28.54 |
| a0033D16 | 22106200 | 22299788 | 16.81 |

Long (L) arm

| Clone name | Start (bp) | End (bp) | Repeat content (%) |
| --- | --- | --- | --- |
| b0002I18 | 29506741 | 29629956 | 11.75 |
| a0041F02 | 29852663 | 30010457 | 20.56 |
| b0011M14 | 30006916 | 30144391 | 26.14 |
| a0037E03 | 31334247 | 31501809 | 28.14 |
| a0036L01 | 31849467 | 32007174 | 15.96 |
| b0007K04 | 34625943 | 34771746 | 18.56 |
| a0009F12 | 36009617 | 36197373 | 27.06 |
| b0032D13 | 36350054 | 36506048 | 18.24 |
| a0014G21 | 36501609 | 36629096 | 21.82 |
| b0011M04 | 36854229 | 37002472 | 5.83 |
| b0025H02 | 37377041 | 37501983 | 7.77 |
| b0035D02 | 37506114 | 37762639 | 19.54 |
| b0040F05 | 37969770 | 38191813 | 21.34 |
| b0040F05 | 38001374 | 38191813 | 22.18 |
| a0037F23 | 39500638 | 39639896 | 26.51 |
| b0047N08 | 40007444 | 40153099 | 19.42 |
| a0009P14 | 40808669 | 40961876 | 29.90 |
| a0047F20 | 40911246 | 41000063 | 9.54 |
| b0036D03 | 41006469 | 41221676 | 17.42 |
| b0015F15 | 41505264 | 41681278 | 7.27 |
| a0013E06 | 42292206 | 42500698 | 12.33 |
| b0039M21 | 42502397 | 42659662 | 27.89 |
| a0033K23 | 42848637 | 43004766 | 13.13 |
| a0043C19 | 43854818 | 44001044 | 17.15 |
| a0038N13 | 44001051 | 44142538 | 18.64 |
| b0016K10 | 44871786 | 45008776 | 24.34 |
| a0023M06 | 45500051 | 45641535 | 23.72 |
| b0040H10 | 46810232 | 47007666 | 25.57 |
| a0005G11 | 47463732 | 47500906 | 8.93 |
| b0018C11 | 47502611 | 47632625 | 20.20 |
| a0043A16 | 48007234 | 48184206 | 19.18 |
| a0028D20 | 48507637 | 48657501 | 20.85 |
| a0026K12 | 48876166 | 49007415 | 8.82 |
| a0026M18 | 49347850 | 49503810 | 4.84 |
| a0018C17 | 49869712 | 50007626 | 7.58 |
| a0019B17 | 50354409 | 50508627 | 7.22 |
| a0025I07 | 50508637 | 50670776 | 9.56 |
| a0037F15 | 50854746 | 51004145 | 4.69 |
| b0009O11 | 51007213 | 51142731 | 21.81 |
| a0041A23 | 51823413 | 52001200 | 28.80 |
| a0018O04 | 52001207 | 52171186 | 16.46 |
| a0023N06 | 52300422 | 52503409 | 11.71 |
| a0037C10 | 52501229 | 52687354 | 9.66 |
| a0024K02 | 53356232 | 53501555 | 12.47 |
| a0030K10 | 54003049 | 54158281 | 4.71 |
| a0028B03 | 54337885 | 54500593 | 16.02 |
| a0002F20 | 54500603 | 54637896 | 3.09 |
| a0008G22 | 55503533 | 55665230 | 5.31 |
| a0043F22 | 56008431 | 56171010 | 19.64 |
| a0044B21 | 56862681 | 57005129 | 9.17 |
| b0034A10 | 57009259 | 57107717 | 7.19 |
| b0037C16 | 57302467 | 57322111 | 1.45 |
| a0008L06 | 57499776 | 57533242 | 7.41 |
| a0020N10 | 57504387 | 57653389 | 5.12 |
