# Supplementary material for: Reconstructing the Evolution of Brachypodium Genomes Using Comparative Chromosome Painting
Source: PLoS One. 2014 Dec 10;9(12):e115108. doi: 10.1371/journal.pone.0115108 (PMC4262448; doi:10.1371/journal.pone.0115108)
Supplement: S4 Table — Characteristics of BAC clones used for the chromosome painting of B. distachyon chromosome 4 (Bd4). (DOCX) [file pone.0115108.s004.docx]

**Table S4.** Characteristics of BAC clones used for the chromosome painting of *B. distachyon* chromosome 4 (Bd4).

Short (S) arm

| Clone name | Start (bp) | End (bp) | Repeat content (%) |
| --- | --- | --- | --- |
| b0022F16 | 1375208 | 1513070 | 17.05 |
| a0029D03 | 1509327 | 1527857 | 14.80 |
| b0011J07 | 1877049 | 2006504 | 13.50 |
| b0030B12 | 2007584 | 2157984 | 7.19 |
| a0008M09 | 3005577 | 3057490 | 10.02 |
| b0031P08 | 4003479 | 4005284 | 0.00 |
| b0020L19 | 4509871 | 4652127 | 17.87 |
| b0015K04 | 5000667 | 5192928 | 12.52 |
| b0015K04 | 5192351 | 5334608 | 20.17 |
| a0021K11 | 5356098 | 5506730 | 12.85 |
| b0040J03 | 7830905 | 8001843 | 19.13 |
| b0021B09 | 9502901 | 9667864 | 12.35 |
| a0039N16 | 9858184 | 10002657 | 14.32 |
| a0043D11 | 11006774 | 11150531 | 22.28 |
| a0038M23 | 13356537 | 13506604 | 23.57 |
| a0004L13 | 13506625 | 13718798 | 11.46 |
| a0010I18 | 14002249 | 14164264 | 10.04 |
| a0028M13 | 14467770 | 14501426 | 10.11 |
| a0006A19 | 15500025 | 15633790 | 10.64 |
| b0023J07 | 18378813 | 18509732 | 28.56 |

Long (L) arm

| Clone name | Start (bp) | End (bp) | Repeat content (%) |
| --- | --- | --- | --- |
| a0047K04 | 27639991 | 27795427 | 18.44 |
| b0012C05 | 28999425 | 29071930 | 12.74 |
| a0006J17 | 29358544 | 29516826 | 9.42 |
| b0027J13 | 30855184 | 31004161 | 28.40 |
| a0013N14 | 31000455 | 31132079 | 9.47 |
| a0020D08 | 32504625 | 32642850 | 8.94 |
| b0033J04 | 32835278 | 33001029 | 24.02 |
| a0029H13 | 33008088 | 33024284 | 20.44 |
| b0005K02 | 33865813 | 34006488 | 20.04 |
| b0002F10 | 34027870 | 34141505 | 10.19 |
| a0021F10 | 35000545 | 35176627 | 26.57 |
| a0011F18 | 36857616 | 37009036 | 26.55 |
| a0031C04 | 37506966 | 37653676 | 24.29 |
| a0004J07 | 38767257 | 38918272 | 6.73 |
| b0021H03 | 38925520 | 39066237 | 14.28 |
| b0035E05 | 39350118 | 39526113 | 6.40 |
| b0014B09 | 39506174 | 39642910 | 17.79 |
| a0024O22 | 39853958 | 40006315 | 16.05 |
| b0041J05 | 40005710 | 40200691 | 17.39 |
| a0032J21 | 40499878 | 40546546 | 5.53 |
| a0003H15 | 40835257 | 41003446 | 12.86 |
| b0047A04 | 41006415 | 41014781 | 22.45 |
| a0047P14 | 41364258 | 41502074 | 13.90 |
| a0026J09 | 41502088 | 41633709 | 24.33 |
| b0032E02 | 41852352 | 42005820 | 21.66 |
| b0026E20 | 42346424 | 42505268 | 15.02 |
| b0038H23 | 42789149 | 43003220 | 6.17 |
| b0023G20 | 43352742 | 43500050 | 9.76 |
| b0019I11 | 43876622 | 44010479 | 6.16 |
| a0004A24 | 44343043 | 44510220 | 7.93 |
| a0043N14 | 45504848 | 45661980 | 6.49 |
| b0043F05 | 46001501 | 46132046 | 15.43 |
| a0030C04 | 46357221 | 46502222 | 7.16 |
| a0004O19 | 46502230 | 46658753 | 9.01 |
| b0031I03 | 46846042 | 47008130 | 20.21 |
| b0026N15 | 47003712 | 47134381 | 8.51 |
| a0017H17 | 47506772 | 47643218 | 6.97 |
| a0024E12 | 48004859 | 48154124 | 3.07 |
| a0041I03 | 48350055 | 48507632 | 9.05 |
