# Supplementary material for: Reconstructing the Evolution of Brachypodium Genomes Using Comparative Chromosome Painting
Source: PLoS One. 2014 Dec 10;9(12):e115108. doi: 10.1371/journal.pone.0115108 (PMC4262448; doi:10.1371/journal.pone.0115108)
Supplement: S5 Table — Characteristics of BAC clones used for the chromosome painting of B. distachyon chromosome 5 (Bd5). (DOCX) [file pone.0115108.s005.docx]

**Table S5.** Characteristics of BAC clones used for the chromosome painting of *B. distachyon* chromosome 5 (Bd5).

Short (S) arm

| Clone name | Start (bp) | End (bp) | Repeat content (%) |
| --- | --- | --- | --- |
| a0018K07 | 853205 | 1009526 | 16.20 |
| a0019O20 | 1091367 | 1236179 | 16.00 |
| b0033P01 | 2501695 | 2512065 | 1.35 |
| a0009O09 | 2504345 | 2648229 | 16.66 |

Long (L) arm

| Clone name | Start (bp) | End (bp) | Repeat content (%) |
| --- | --- | --- | --- |
| a0045F23 | 13499779 | 13653343 | 18.89 |
| b0030K21 | 15304885 | 15505515 | 22.56 |
| a0026M04 | 17499731 | 17633162 | 23.72 |
| a0023L21 | 17634500 | 17679830 | 4.89 |
| a0001F13 | 17802975 | 18003203 | 23.52 |
| a0017D24 | 18003221 | 18155770 | 16.96 |
| b0042J14 | 18312982 | 18503857 | 19.62 |
| a0046O09 | 20358624 | 20503060 | 24.99 |
| b0024J19 | 20845837 | 21003148 | 11.02 |
| b0033K07 | 21003184 | 21110356 | 11.50 |
| b0037B05 | 21507488 | 21710347 | 23.77 |
| b0016H11 | 21877774 | 22006306 | 16.69 |
| b0041K21 | 23480858 | 23500119 | 1.39 |
| b0032J06 | 23870997 | 24003288 | 6.19 |
| a0045J11 | 24003128 | 24154997 | 9.30 |
| a0026B16 | 24841254 | 25002060 | 5.96 |
| a0031B15 | 25503136 | 25695070 | 4.94 |
| a0023B07 | 25746075 | 25906029 | 8.99 |
| a0019J13 | 25906054 | 26098440 | 3.35 |
